# Supplementary figures and images for: HMGCR inhibition stabilizes the glycolytic enzyme PKM2 to support the growth of renal cell carcinoma
Source: PLoS Biol. 2021 Apr 27;19(4):e3001197. doi: 10.1371/journal.pbio.3001197 (PMC8104400; doi:10.1371/journal.pbio.3001197)

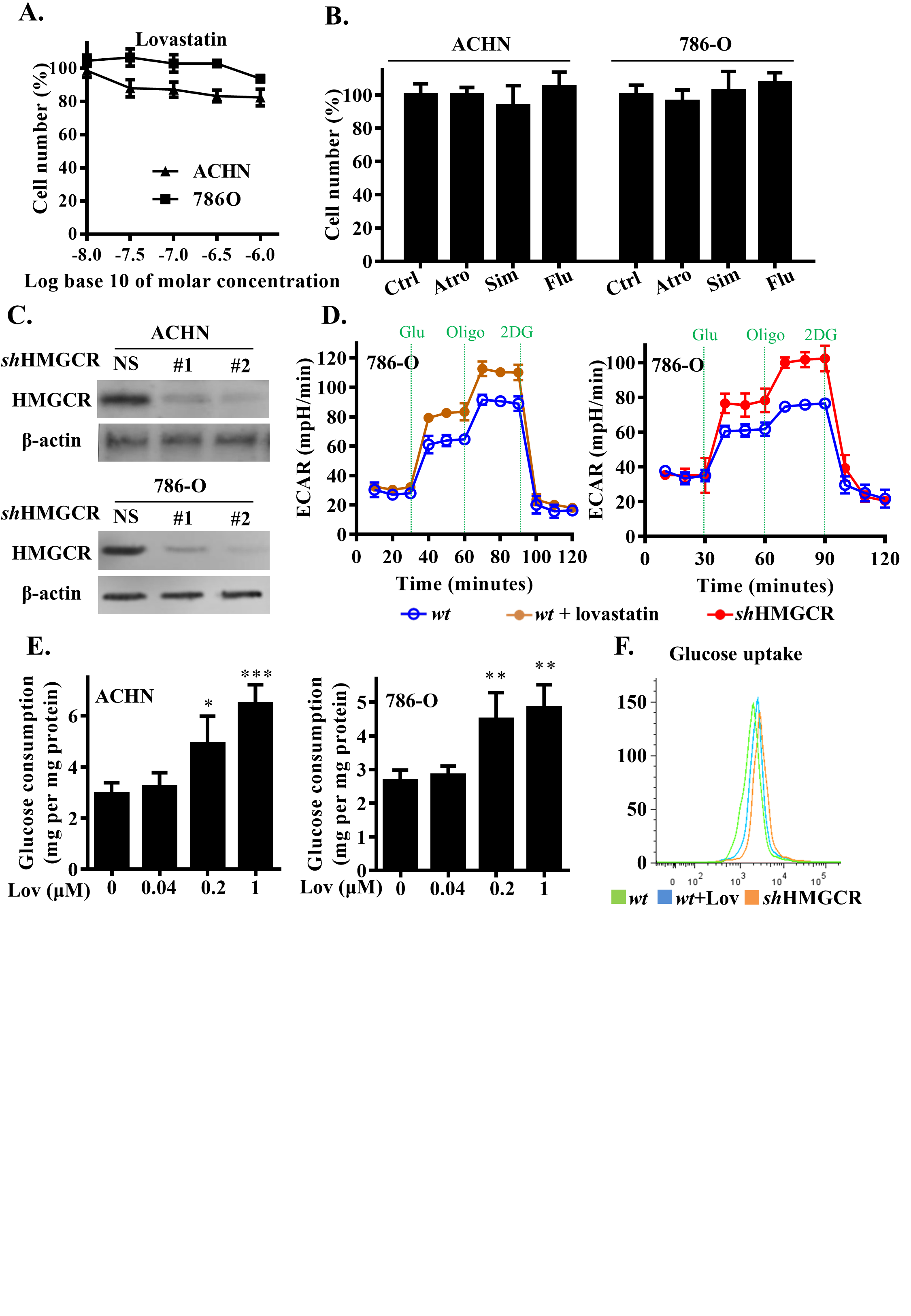

Supplement: S1 Fig — (A) Dose-dependent lovastatin treated on the proliferation of ACHN and 786-O cells. (B) Cell proliferation analysis in ACHN and 786-O cells treated with atorvastatin (Atr), simvastatin (Sim), or fluvastatin (Flu). (C) Western blotting analysis of HMGCR after lentiviral knockdown as exemplified in ACHN and 786-O cells. (D) Kinetic ECAR response of HMGCR knockdown or lovastatin-treated 786-O cells. (E) Glucose consumption of ACHN or 786-O cells under 24-hour lovastatin treatment. (F) Intracellular 2-NBDG glucose uptake in lovastatin-treated cells was analyzed by flow cytometry. The data are represented as the mean ± SD from 3 independent experiments, *(p ≤ 0.05), **(p ≤ 0.01), or ***(p ≤ 0.001). 2DG, 2-deoxy-D-glucose; Atr, atorvastatin; Ctrl, control; ECAR, extracellular acidification rate; Flu, fluvastatin; Glu, glucose; HMGCR, 3-hydroxy-3-methylglutaryl coenzyme A reductase; Lov, lovastatin; NS, non-specific; Oligo, oligomycin; RCC, renal cell carcinoma; Sim, simvastatin; wt, wild type. (TIF) [file pbio.3001197.s001.TIF]

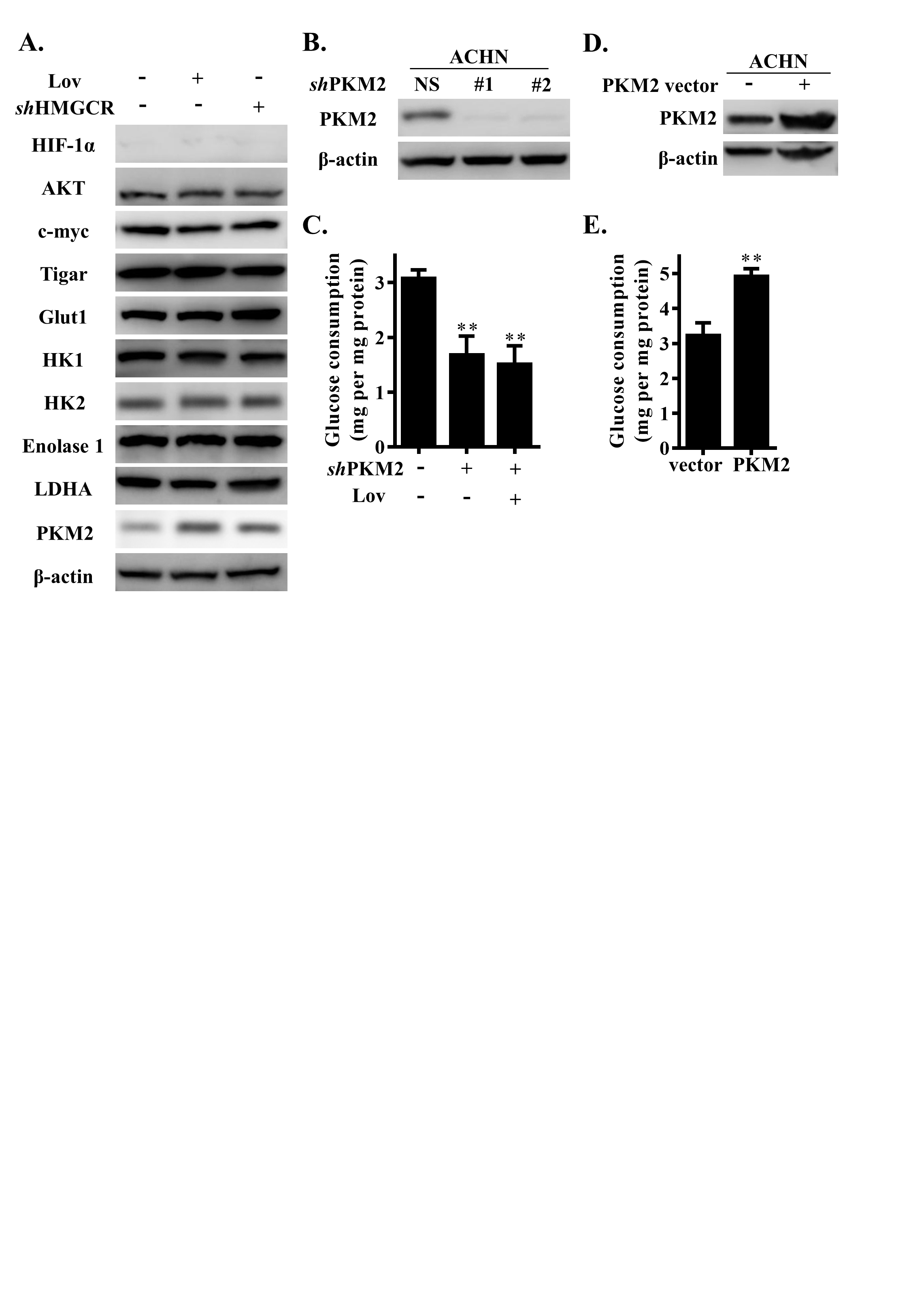

Supplement: S2 Fig — (A) The effect of lovastatin or shHMGCR intervention on the protein levels of glycolysis-related enzymes. (B) Western blotting analysis of PKM2 after lentiviral knockdown as exemplified in ACHN cells. (C) Glucose consumption in PKM2 knockdown ACHN cells with or without lovastatin treatment. (D) Western blotting analysis of PKM2 with exogenous overexpression as exemplified in ACHN cells. (E) Glucose consumption in PKM2 overexpression ACHN cells. The data are represented as the mean ± SD from 3 independent experiments, *(p ≤ 0.05), **(p ≤ 0.01), or ***(p ≤ 0.001). Glut1, glucose transporter 1; HIF-1α, hypoxia-inducible factor-1α; HMGCR, 3-hydroxy-3-methylglutaryl coenzyme A reductase; LDHA, lactate dehydrogenase A; Lov, lovastatin; NS, non-specific; PKM2, pyruvate kinase M2. (TIF) [file pbio.3001197.s002.TIF]

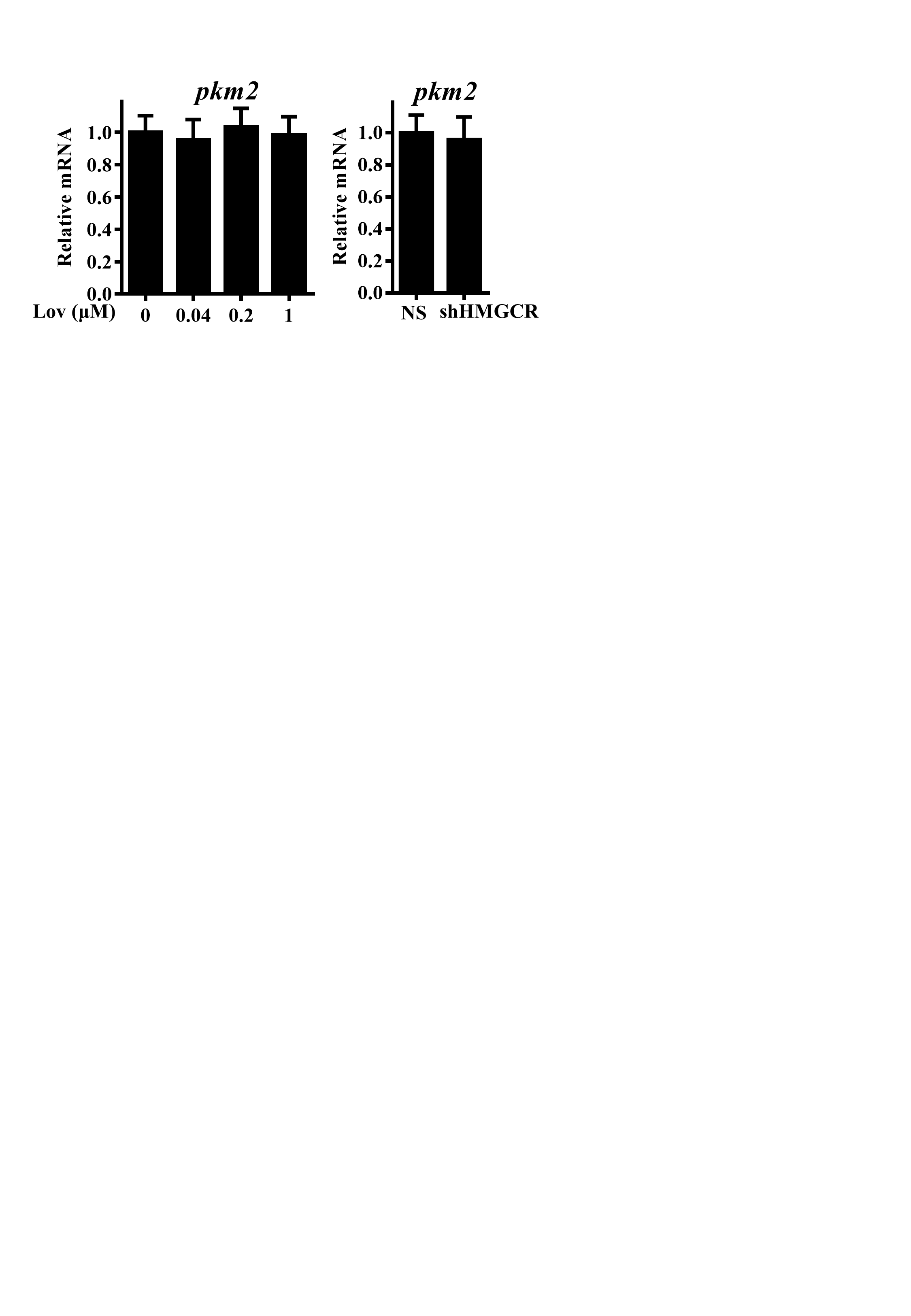

Supplement: S3 Fig — pkm2 expression level in lovastatin-treated (left panel) or HMGCR knockdown (right panel) ACHN cells. The data are represented as the mean ± SD from 3 independent experiments. HMGCR, 3-hydroxy-3-methylglutaryl coenzyme A reductase; Lov, lovastatin; NS, non-specific; pkm2, pyruvate kinase M2. (TIF) [file pbio.3001197.s003.TIF]

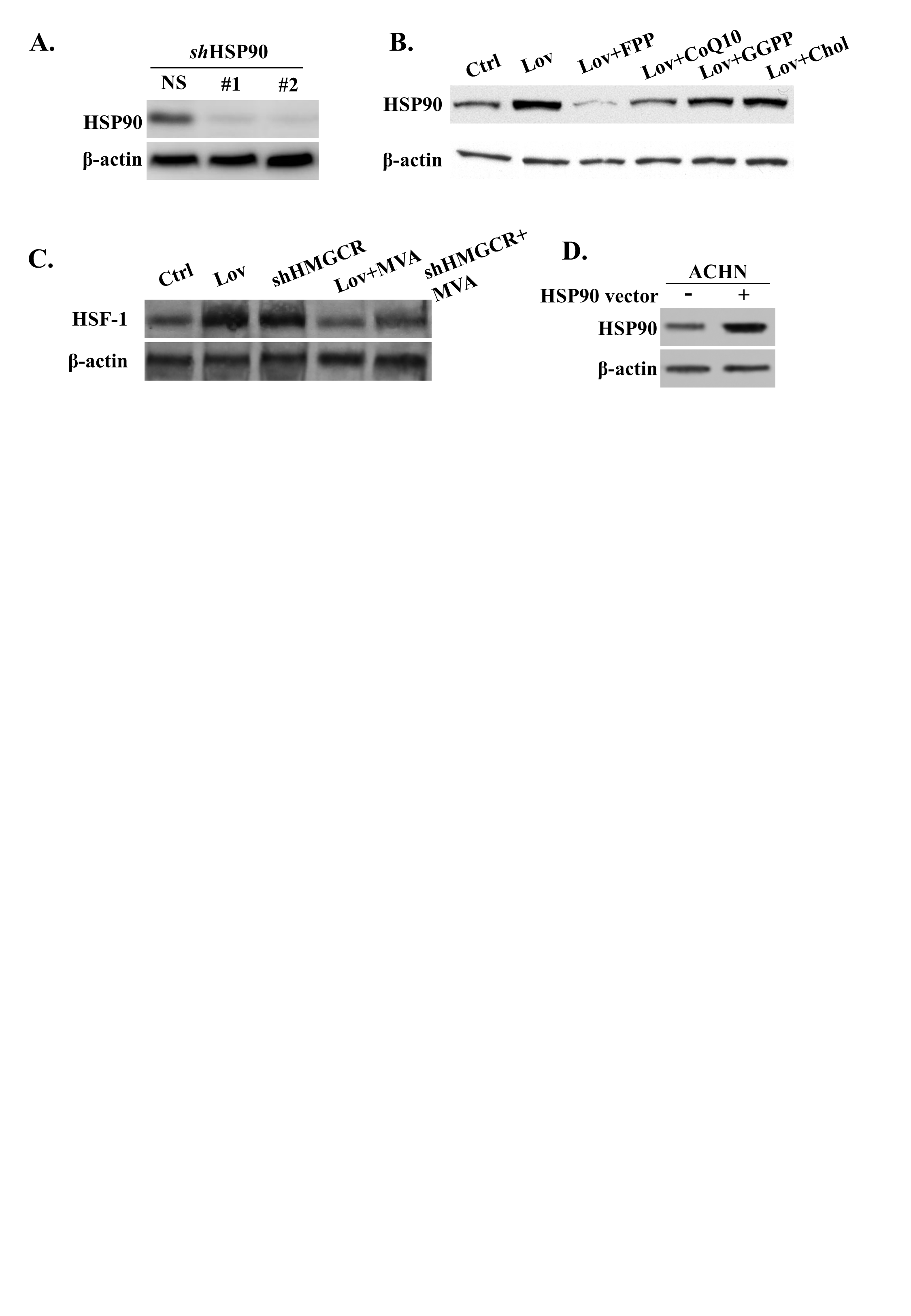

Supplement: S4 Fig — (A) Western blotting analysis of HSP90 after lentiviral knockdown as exemplified in ACHN cells. (B) Supplemental effect of MVA pathway downstream metabolites on HSP90 level in lovastatin-treated ACHN cells. (C) Lovastatin and shHMGCR intervention increases the level of HSF-1 protein, and the effect is rescued when mevalonate is supplemented. (D) Western blotting analysis of HSP90 with exogenous overexpression as exemplified in ACHN cells. Chol, cholesterol; CoQ10, coenzyme Q10; Ctrl, control; FPP, farnesyl pyrophosphate; GGPP, geranylgeranyl pyrophosphate; HMGCR, 3-hydroxy-3-methylglutaryl coenzyme A reductase; HSF-1, heat shock transcription factor-1; HSP90, heat shock protein 90; Lov, lovastatin; MVA, mevalonate; NS, non-specific. (TIF) [file pbio.3001197.s004.TIF]

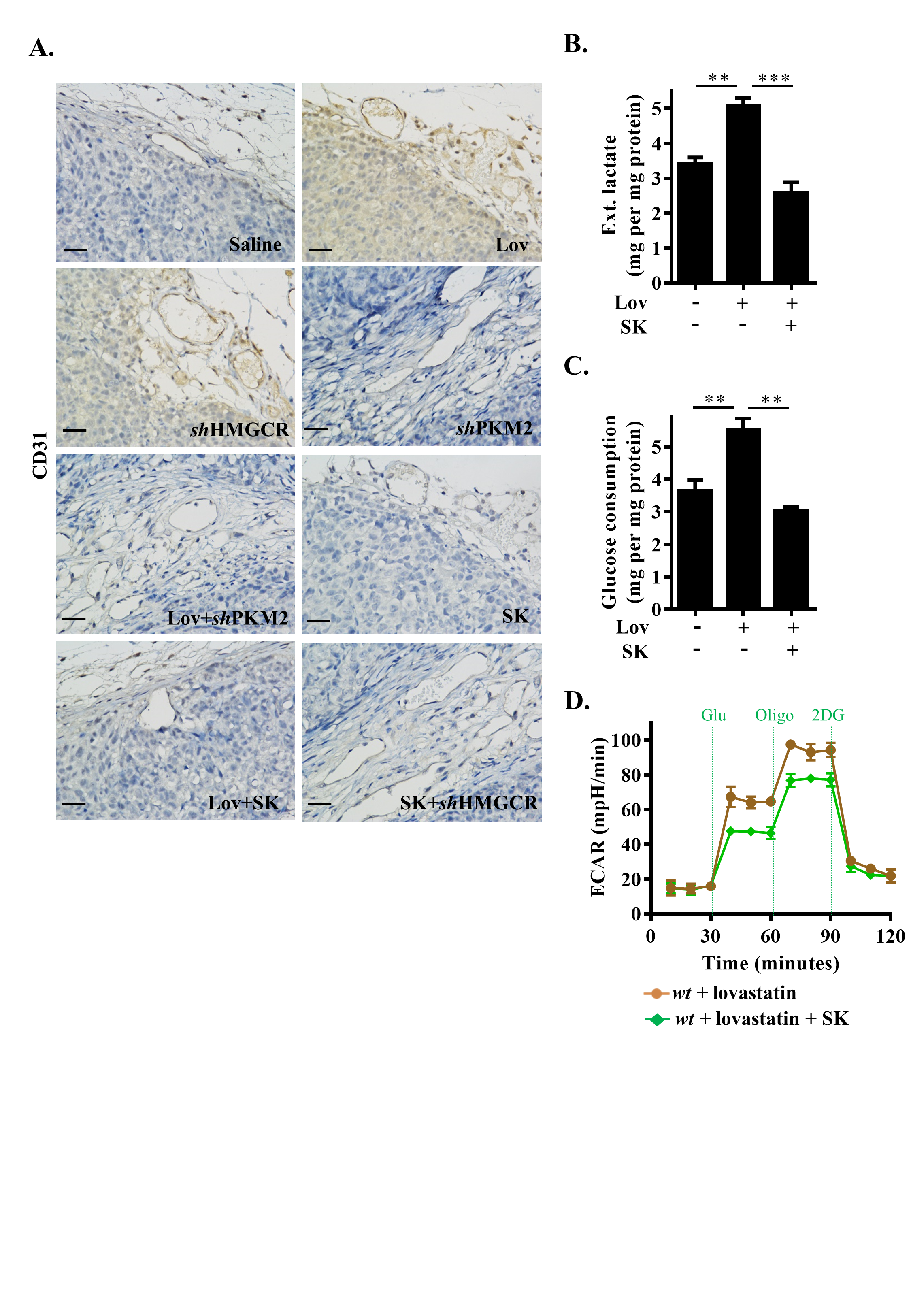

Supplement: S5 Fig — (A) Immunohistochemical staining of CD31+ in ACHN cell xenografts (scale bar: 50 μm). (B) Extracellular lactate level, (C) glucose consumption, and (D) ECAR rate of ACHN cell with or without lovastatin and Shikonin treatment. The data are represented as the mean ± SD from 3 independent experiments, *(p ≤ 0.05), **(p ≤ 0.01) or ***p ≤ 0.001). 2DG, 2-deoxy-D-glucose; ECAR, extracellular acidification rate; Glu, glucose; HMGCR, 3-hydroxy-3-methylglutaryl coenzyme A reductase; Lov, lovastatin; Oligo, oligomycin; PKM2, pyruvate kinase M2; RCC, renal cell carcinoma; SK, Shikonin; wt, wild type. (TIF) [file pbio.3001197.s005.TIF]
