# Supplementary material for: HMGCR inhibition stabilizes the glycolytic enzyme PKM2 to support the growth of renal cell carcinoma
Source: PLoS Biol. 2021 Apr 27;19(4):e3001197. doi: 10.1371/journal.pbio.3001197 (PMC8104400; doi:10.1371/journal.pbio.3001197)

Figure 1A

Saline Group

Lovastatin Group

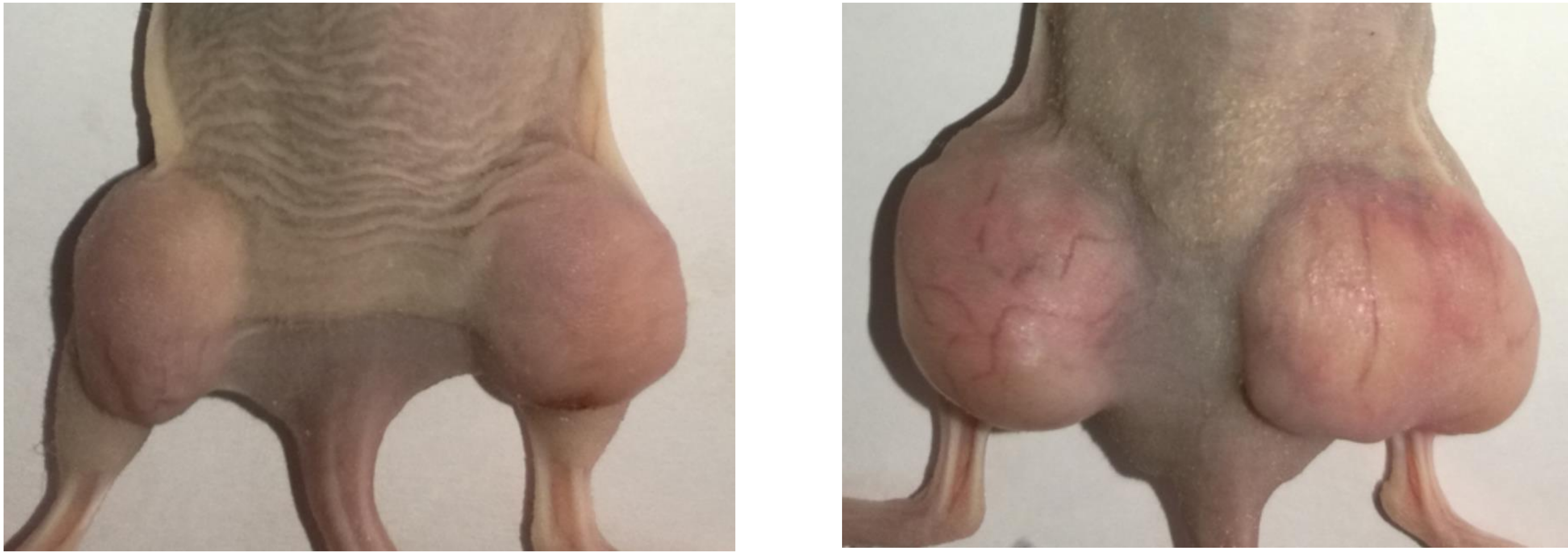

Figure 1H

Saline Group

Lovastatin Group

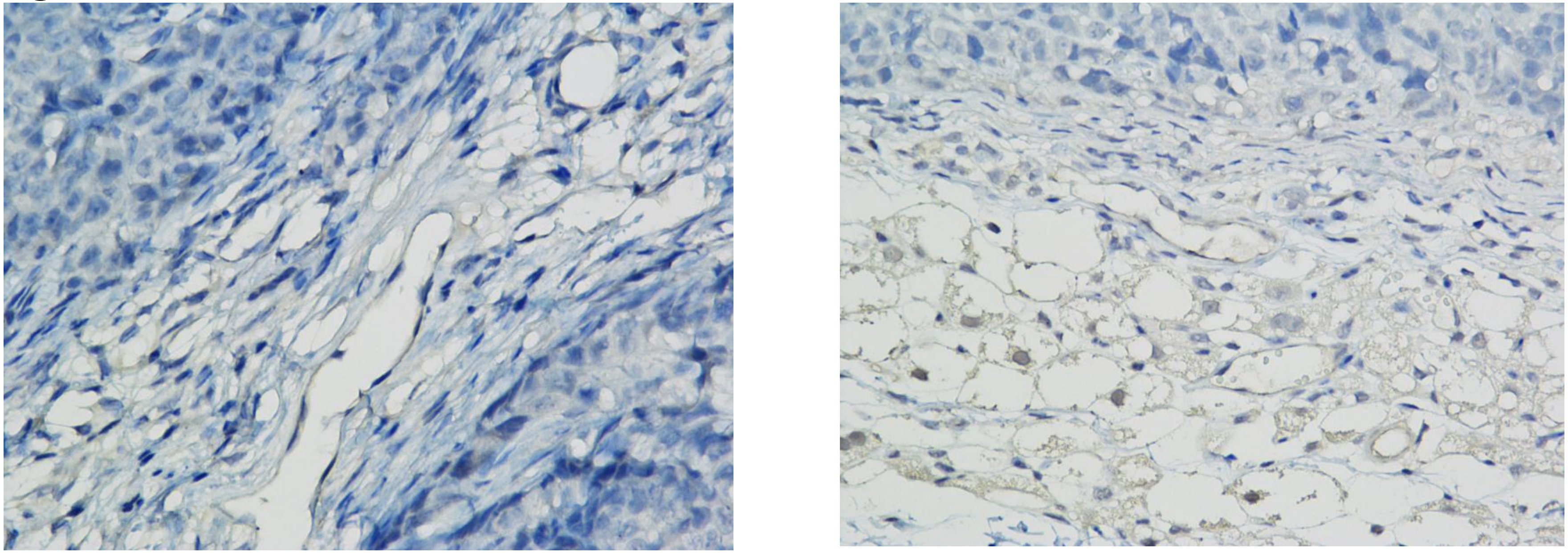

Figure 3A

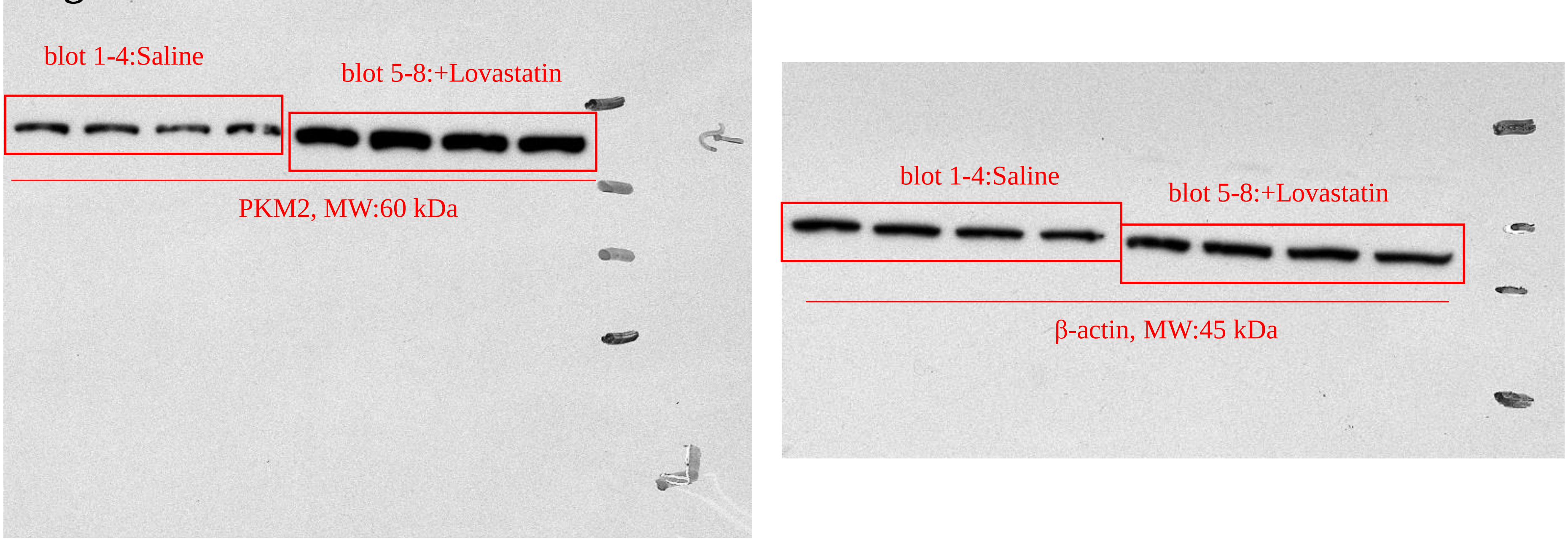

Figure 3B

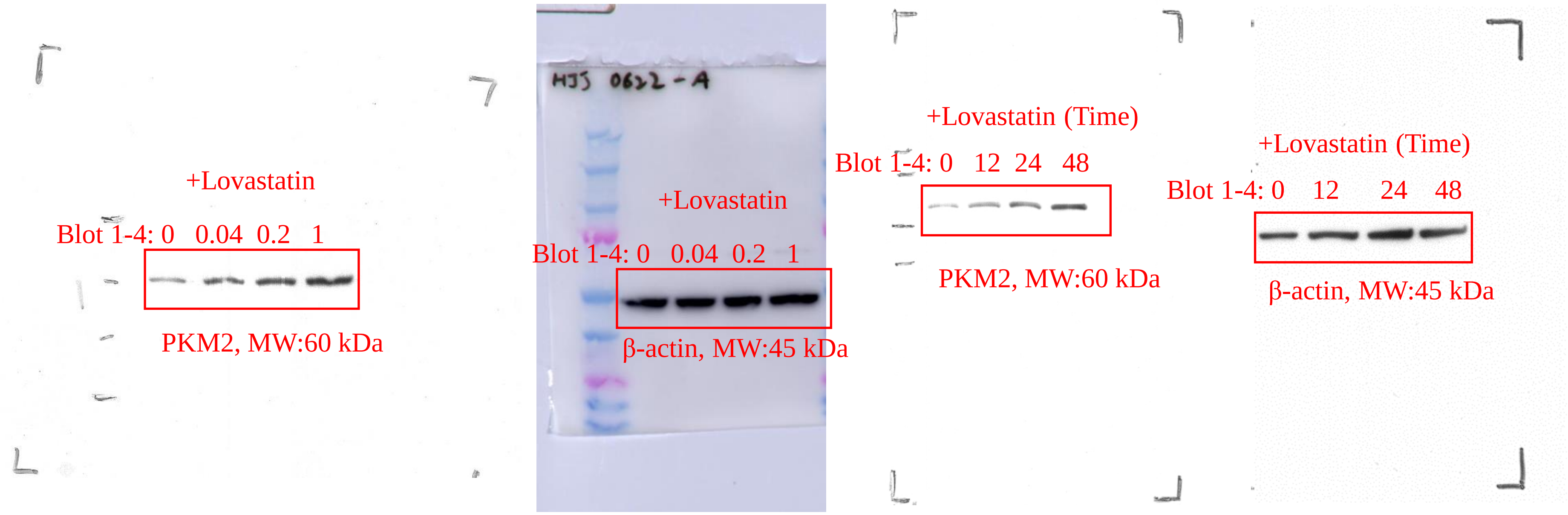

Figure 3E

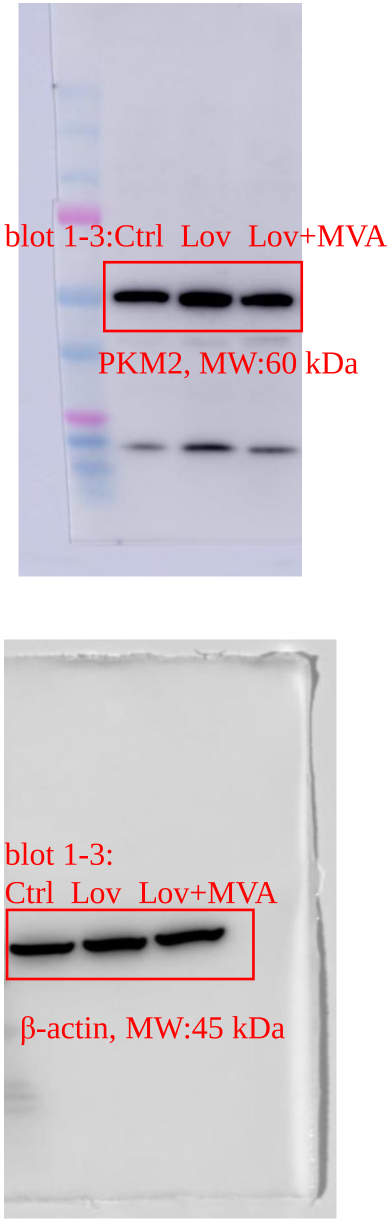

Figure 3J

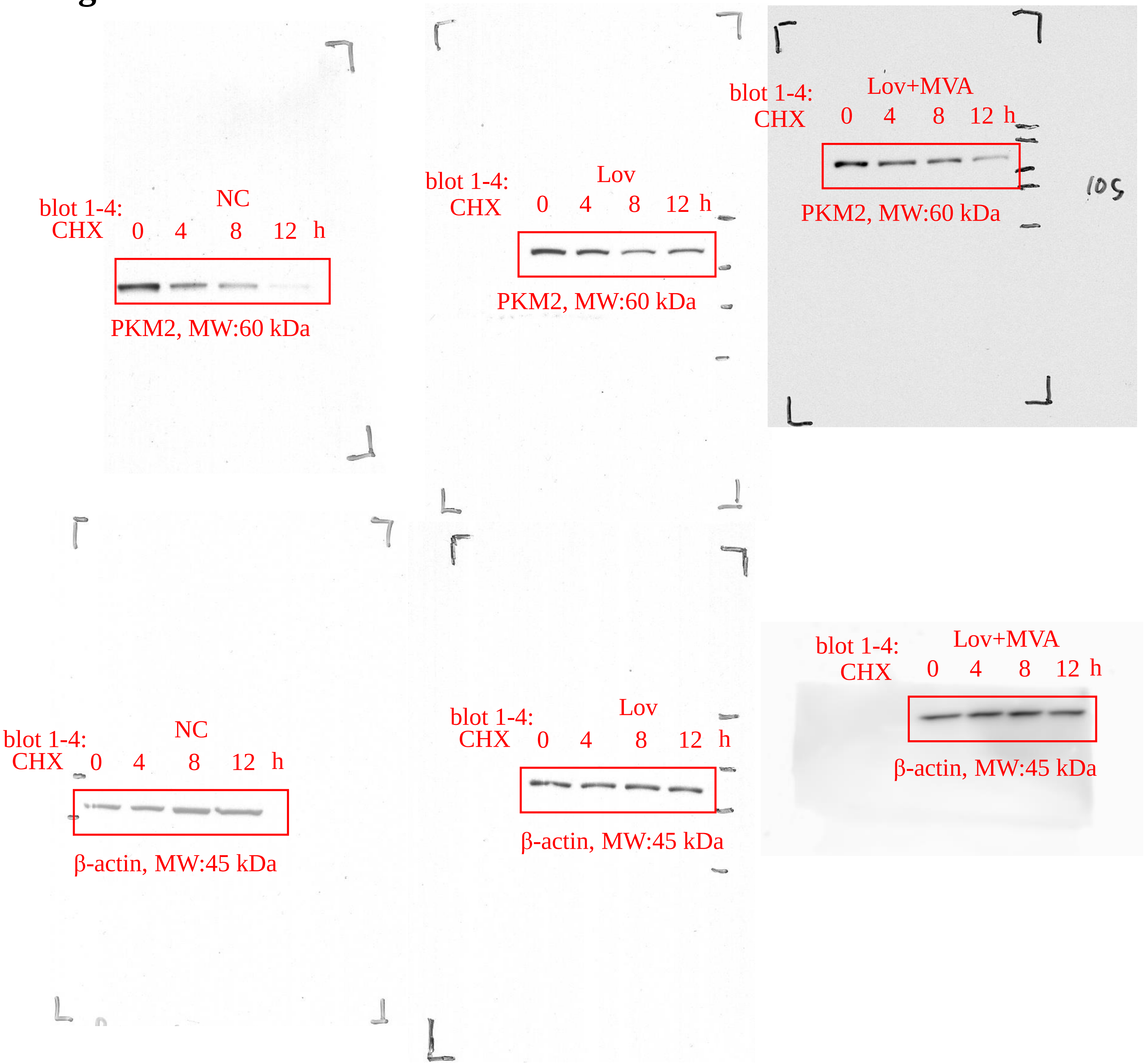

Figure 3K

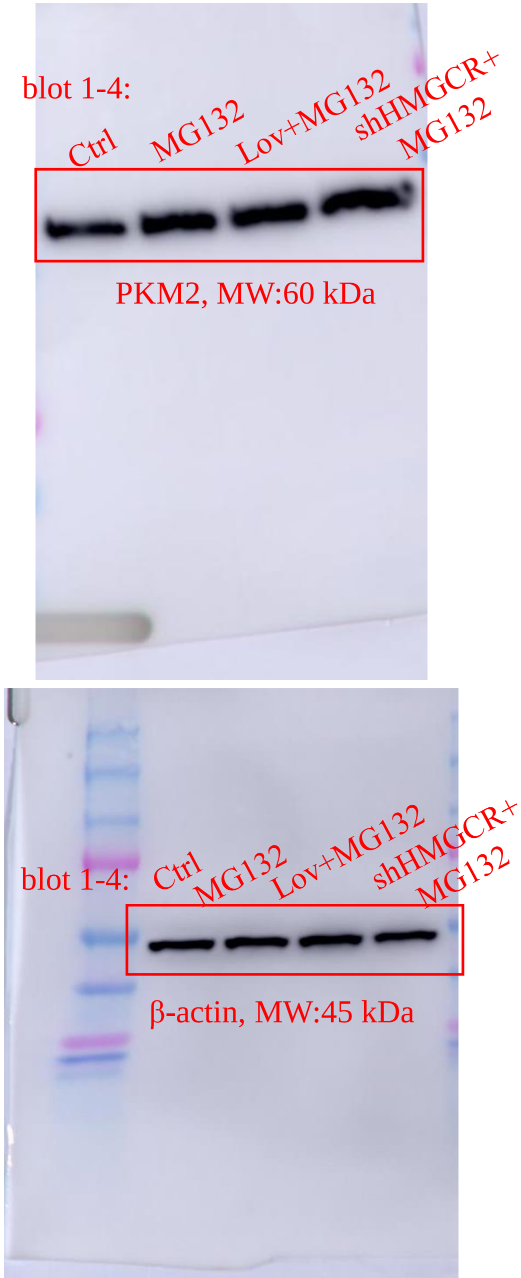

Figure 4A

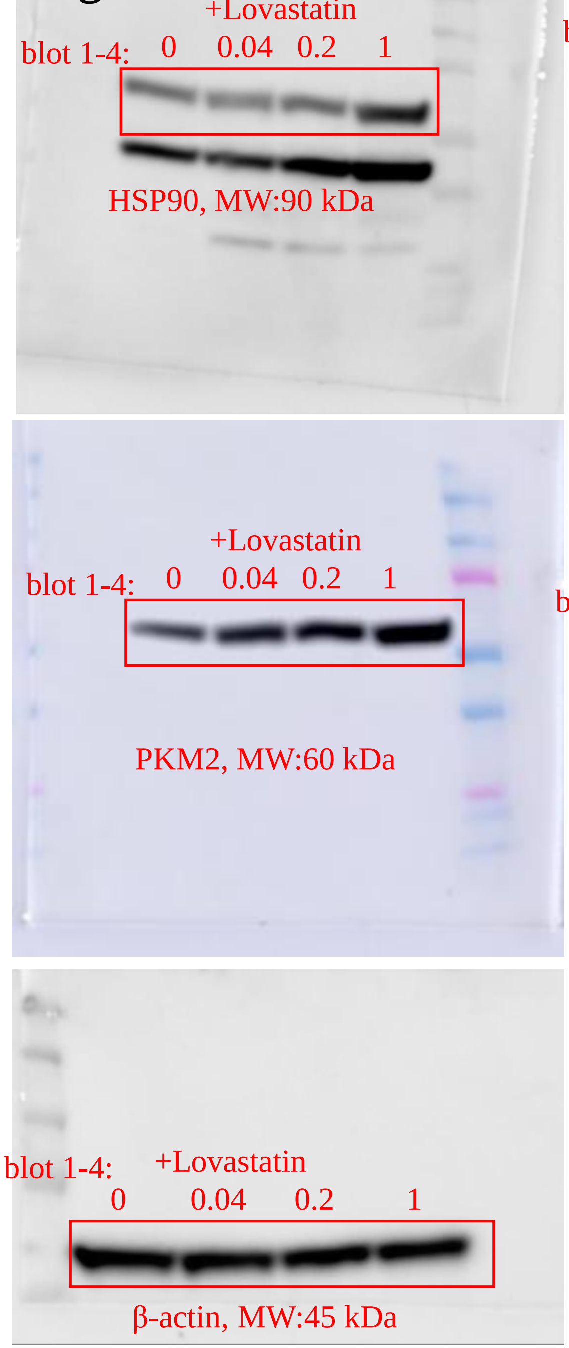

Figure 4B

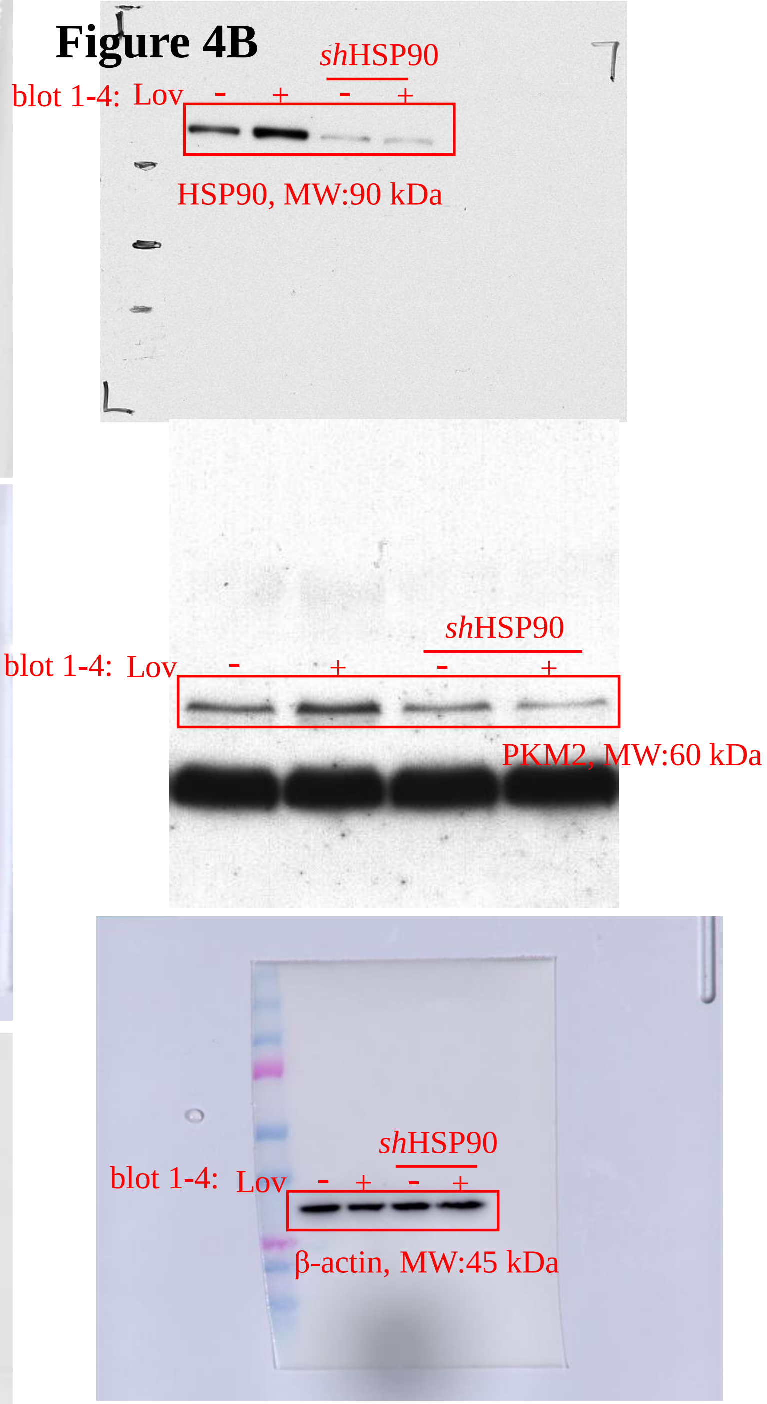

Figure 4C

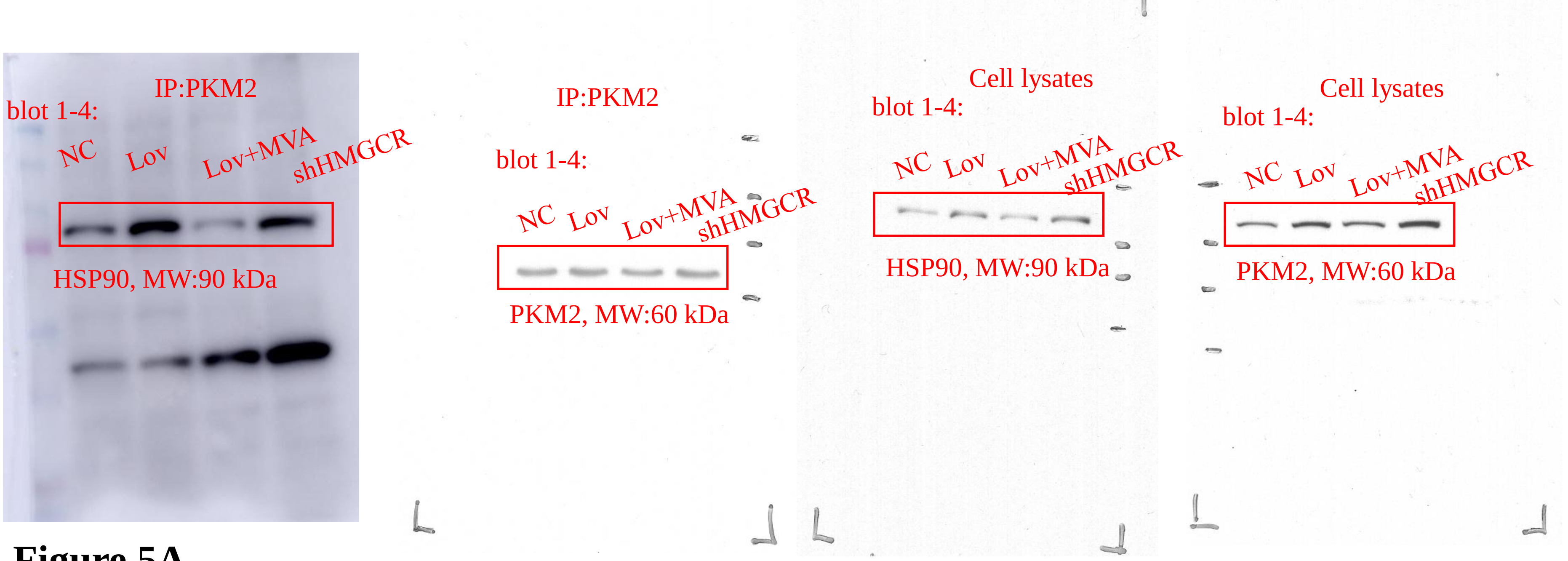

Figure 5A

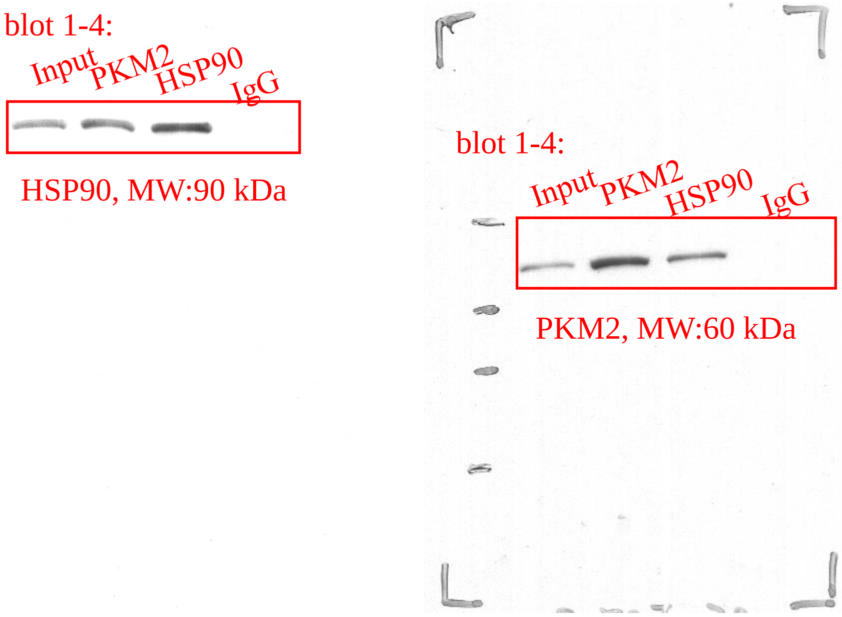

Figure 5B

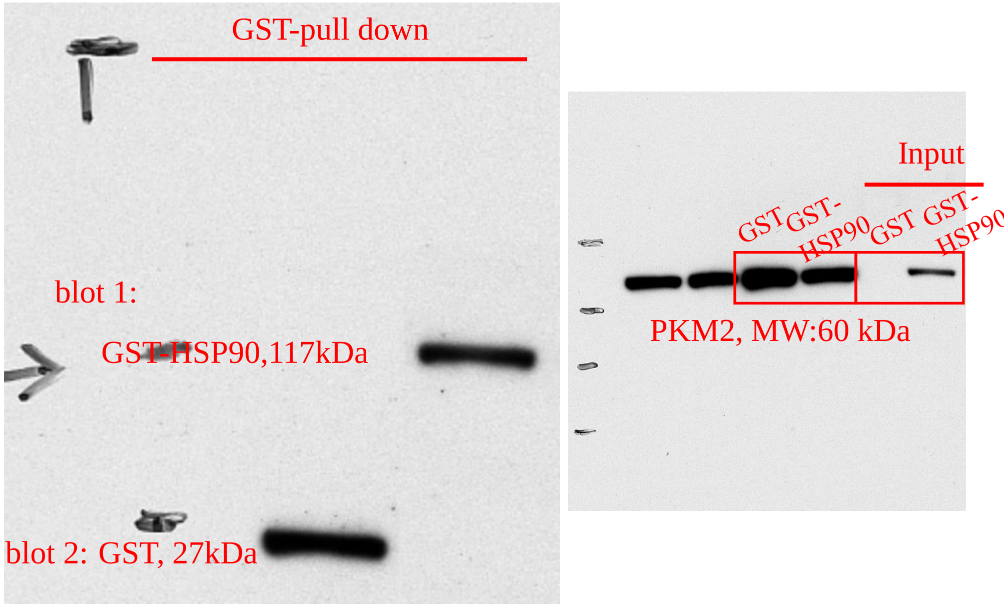

Figure 5C

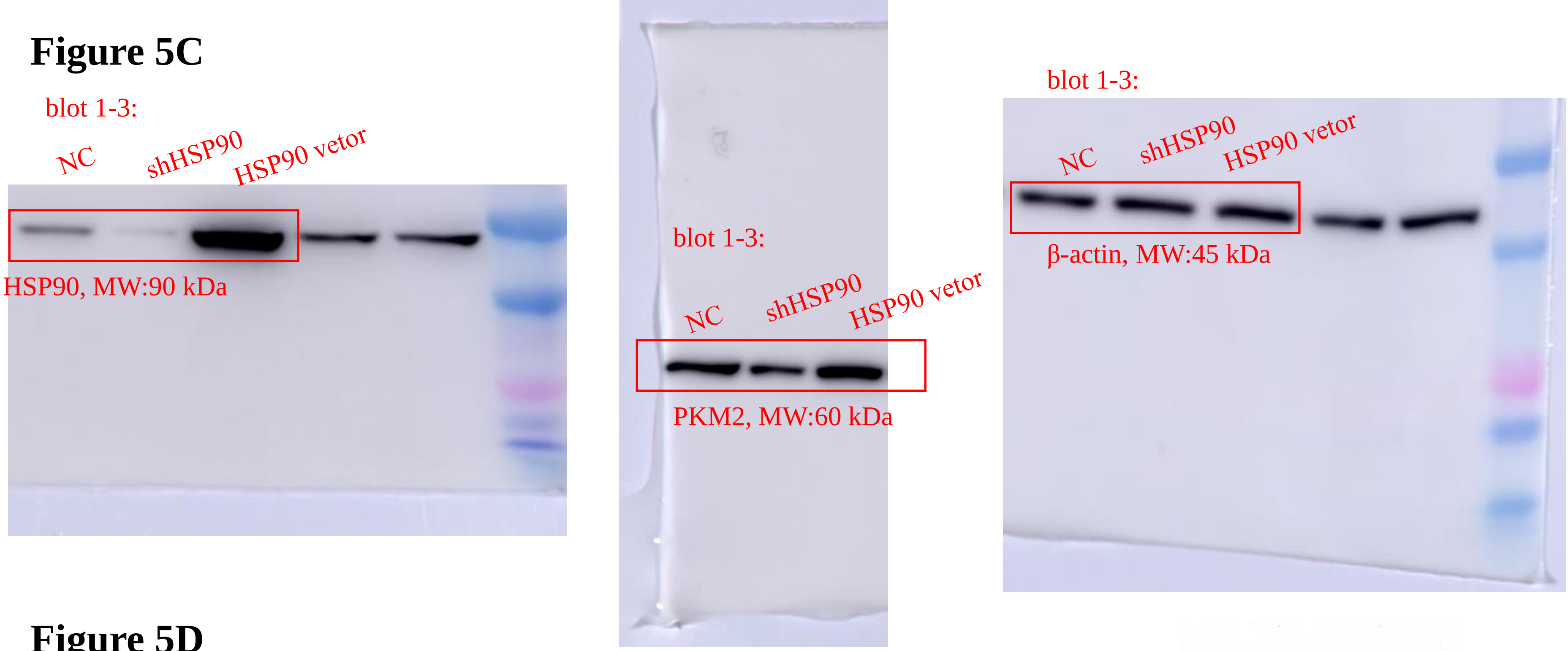

Figure 5D

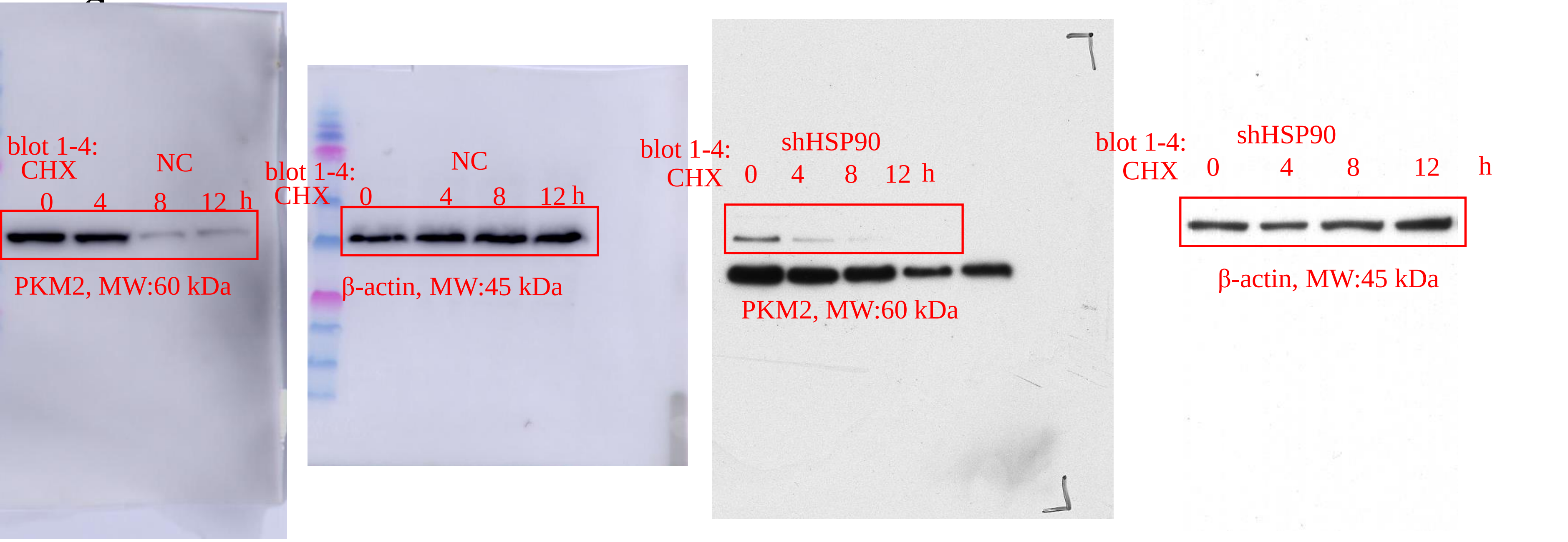

Figure 5D

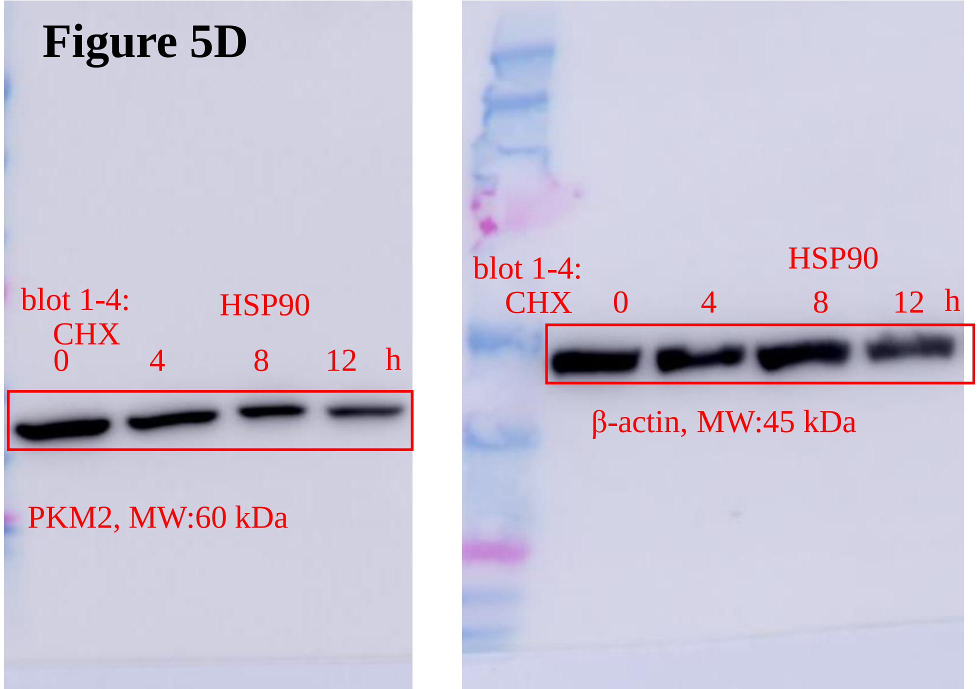

Figure 5E

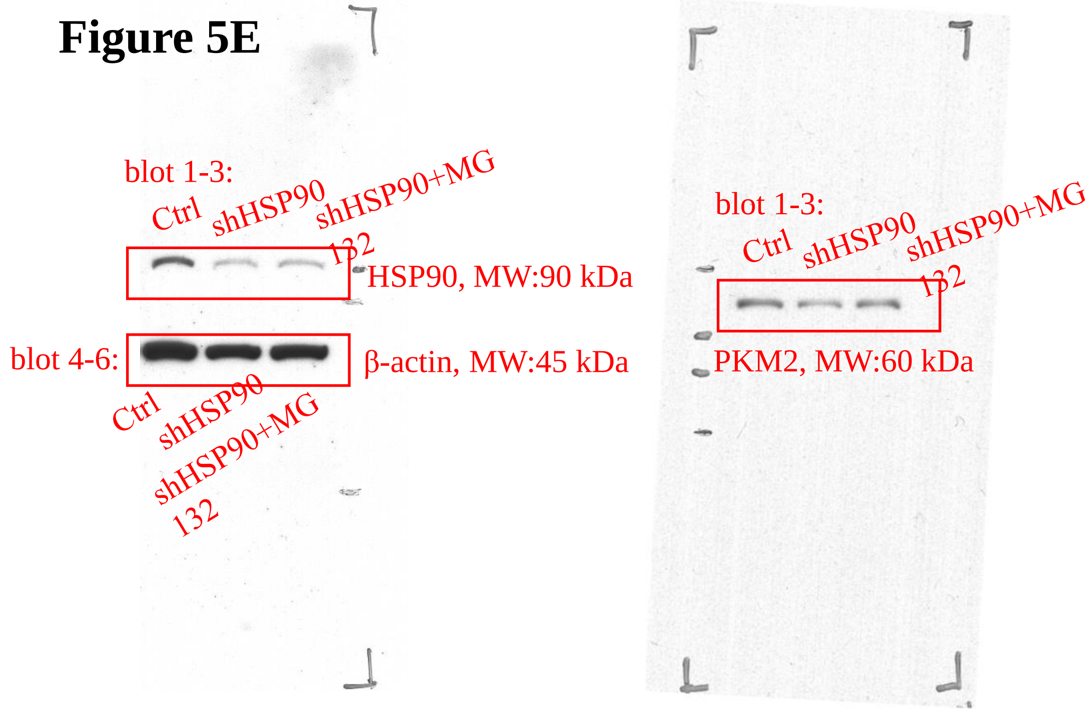

Figure 5F

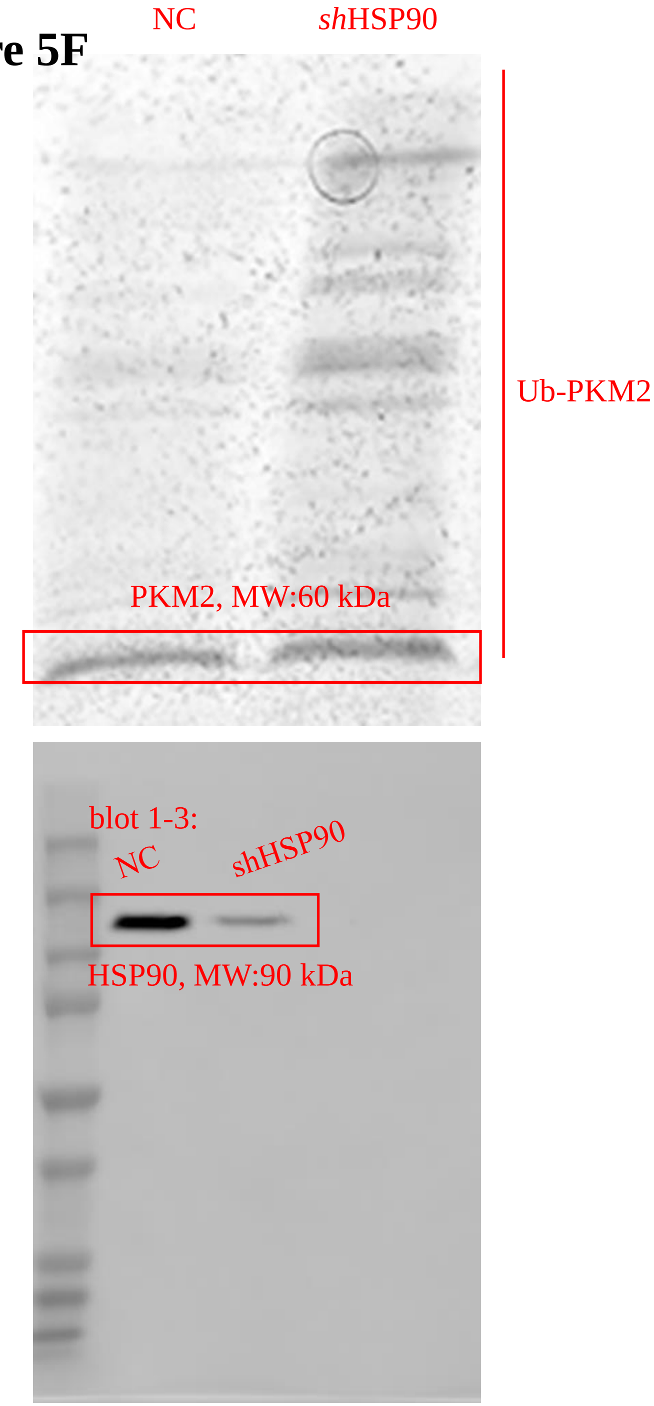

Figure S1.C

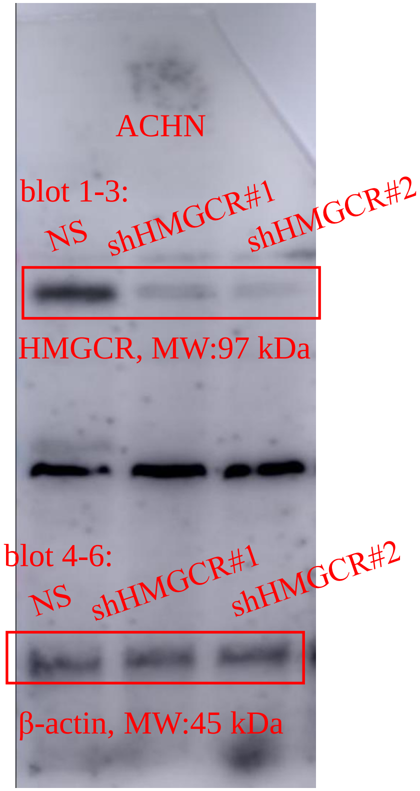

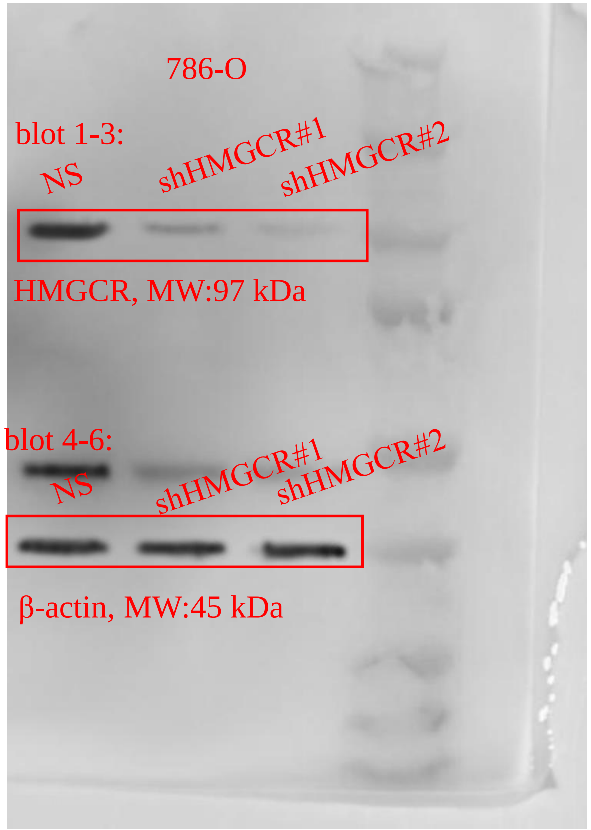

Figure S1.F

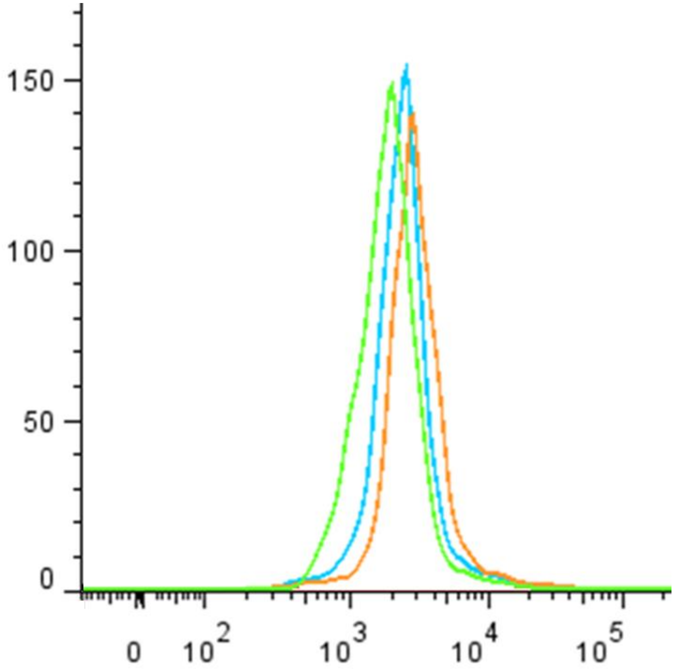

Figure S1.F

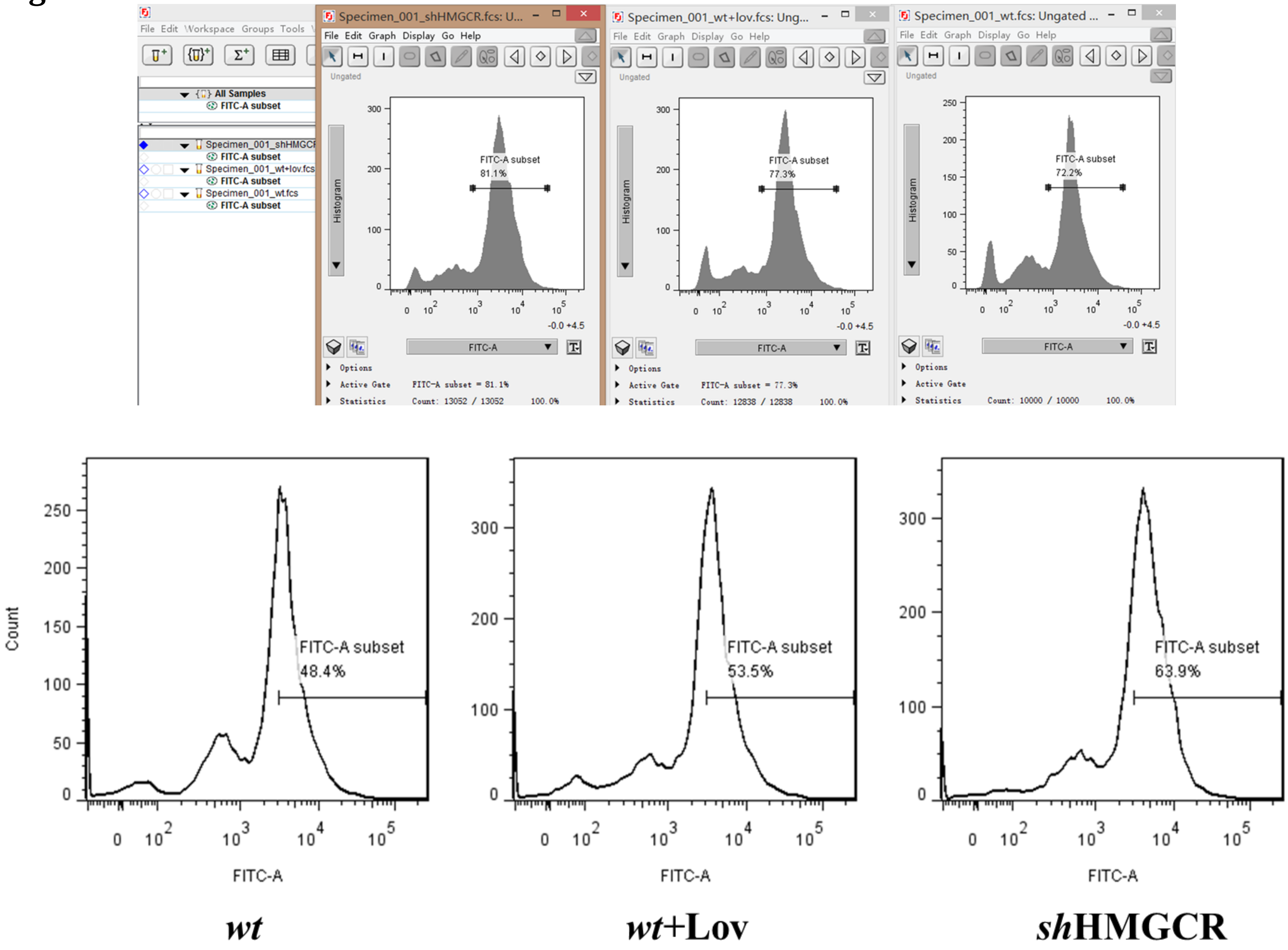

Specimen_001_shHMGCR.fcs: U...
Specimen_001_wt+lov.fcs: Ung...
Specimen_001_wt.fcs: Ungated ...
File Edit Workspace Groups Tools
File Edit Graph Display Go Help
Ungated
All Samples
FITC-A subset
Specimen_001_shHMGCR
Specimen_001_wt+lov.fcs
Specimen_001_wt.fcs
Histogram
FITC-A subset
81.1%
77.3%
72.2%
FITC-A
Options
Active Gate
Statistics
FITC-A subset = 81.1%
FITC-A subset = 77.3%
Count: 13052 / 13052 100.0%
Count: 12838 / 12838 100.0%
Count: 10000 / 10000 100.0%
-0.0 +4.5
Count
FITC-A subset
48.4%
53.5%
63.9%
FITC-A
wt
wt+Lov
shHMGCR

Figure S2.A

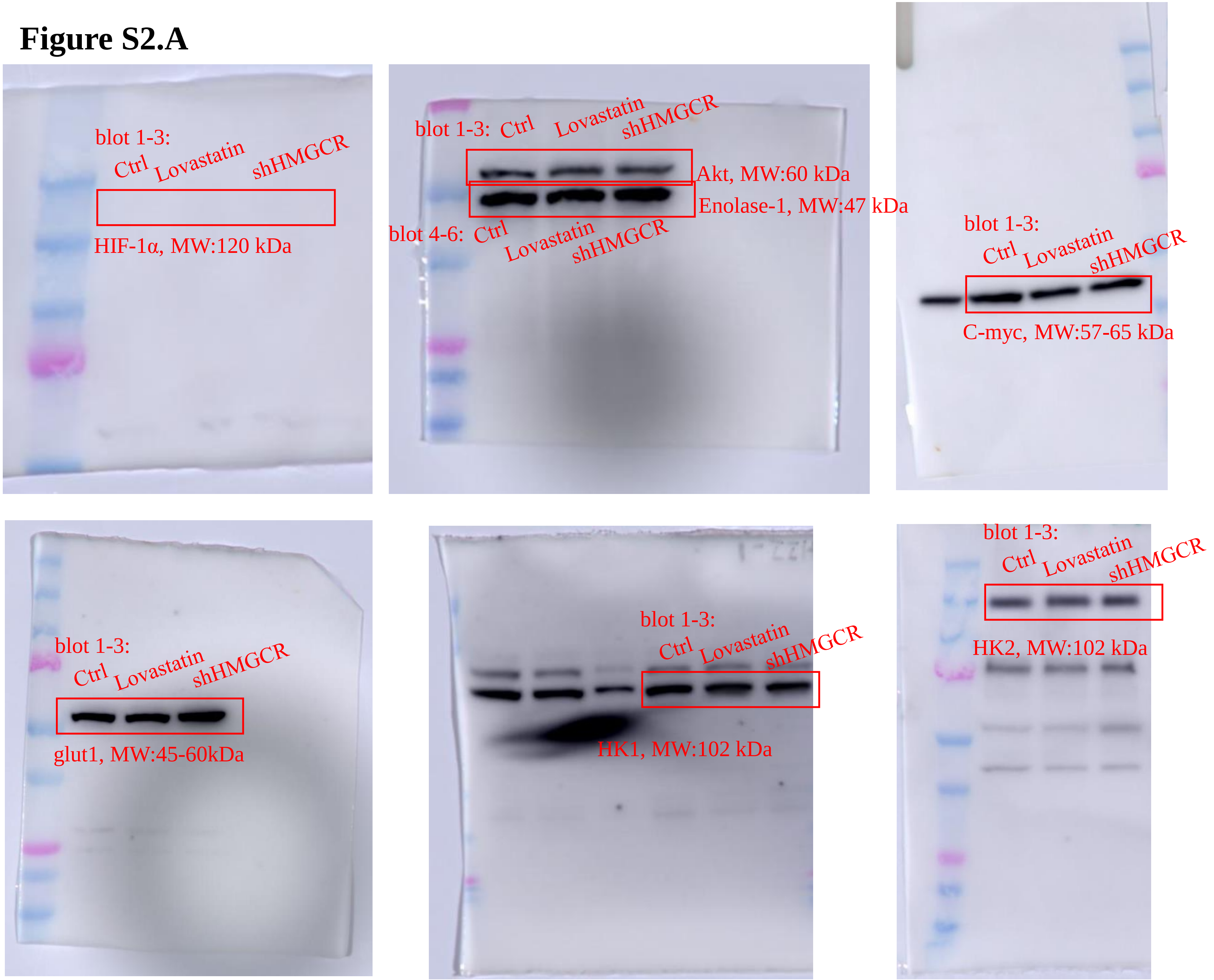
blot 1-3:
Ctrl
Lovastatin
shHMGCR
HIF-1α, MW:120 kDa
blot 1-3: Ctrl
Lovastatin
shHMGCR
Akt, MW:60 kDa
Enolase-1, MW:47 kDa
blot 4-6: Ctrl
Lovastatin
shHMGCR
blot 1-3:
Ctrl
Lovastatin
shHMGCR
C-myc, MW:57-65 kDa
blot 1-3:
Ctrl
Lovastatin
shHMGCR
glut1, MW:45-60kDa
blot 1-3:
Ctrl
Lovastatin
shHMGCR
HK1, MW:102 kDa
blot 1-3:
Ctrl
Lovastatin
shHMGCR
HK2, MW:102 kDa

Figure S2.A

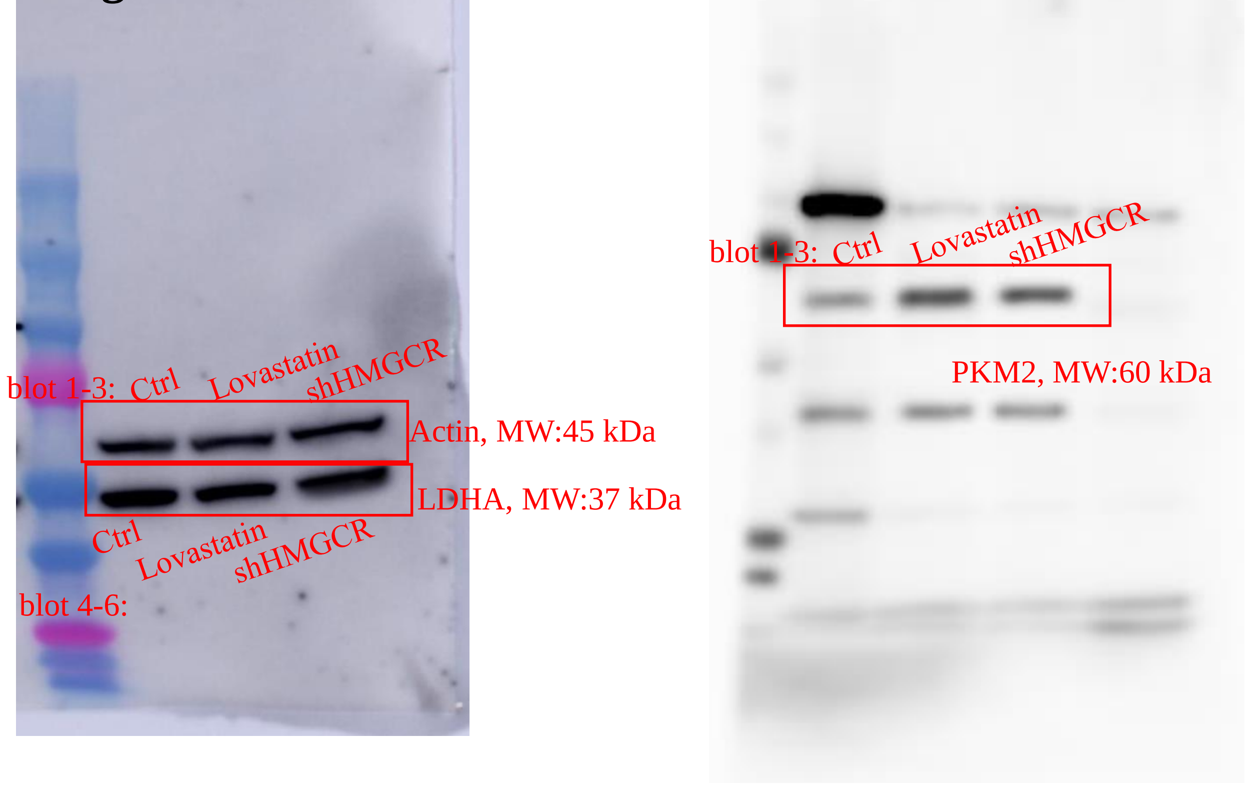

Figure S2.D

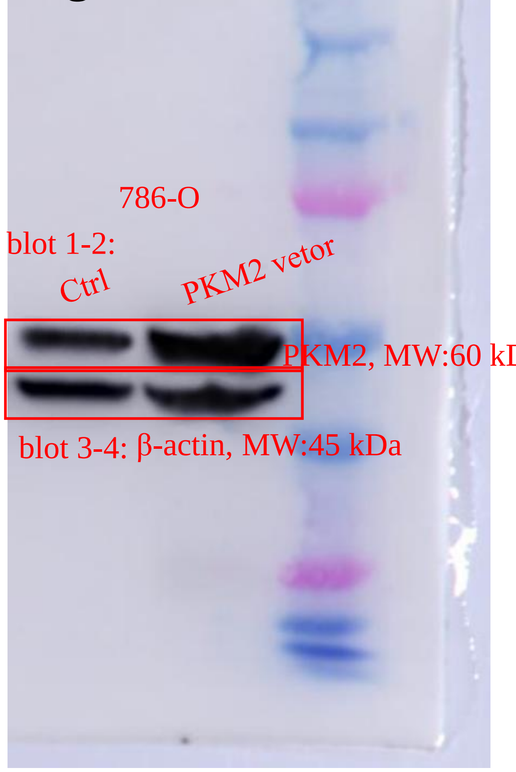

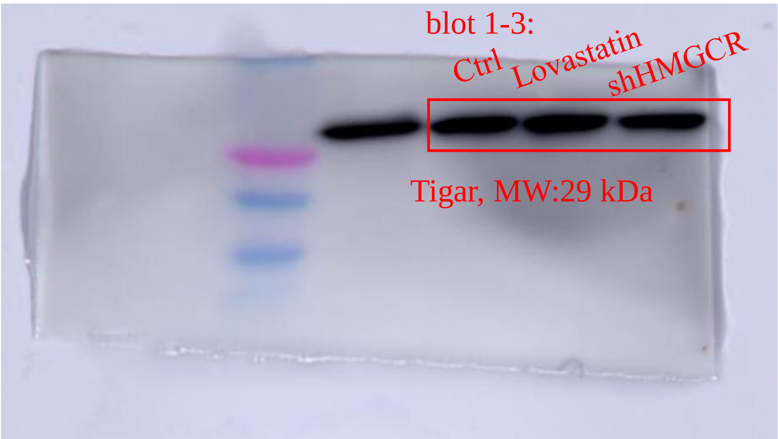

Figure S2.B

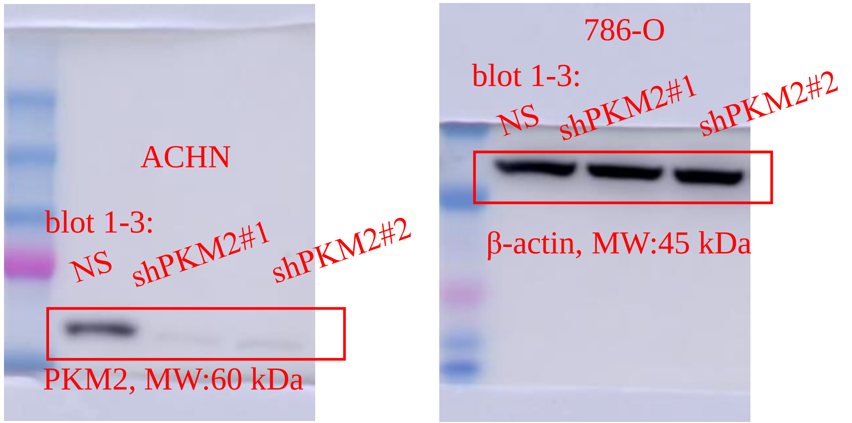

Figure S4.A

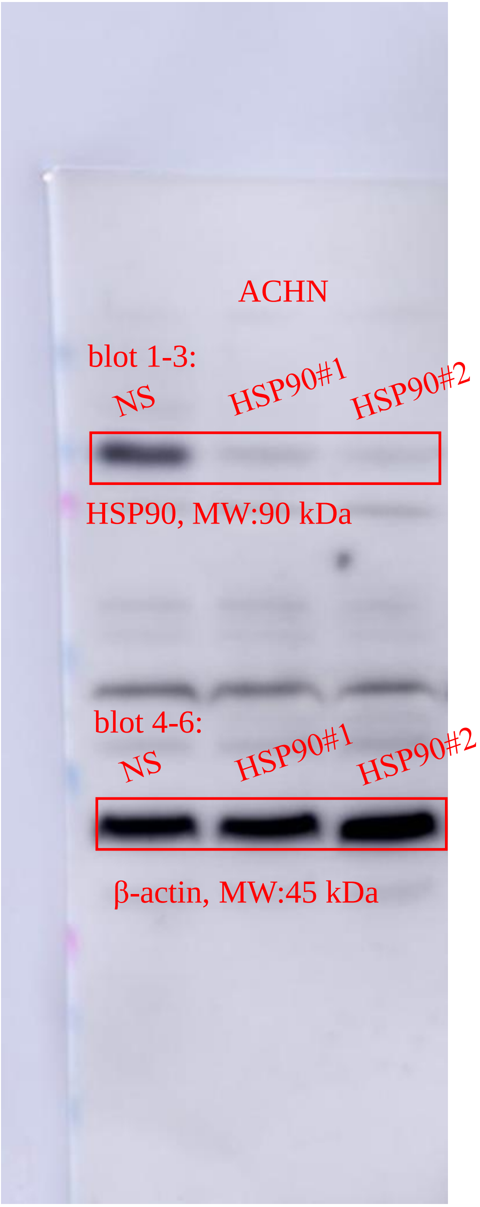

Figure S4.B

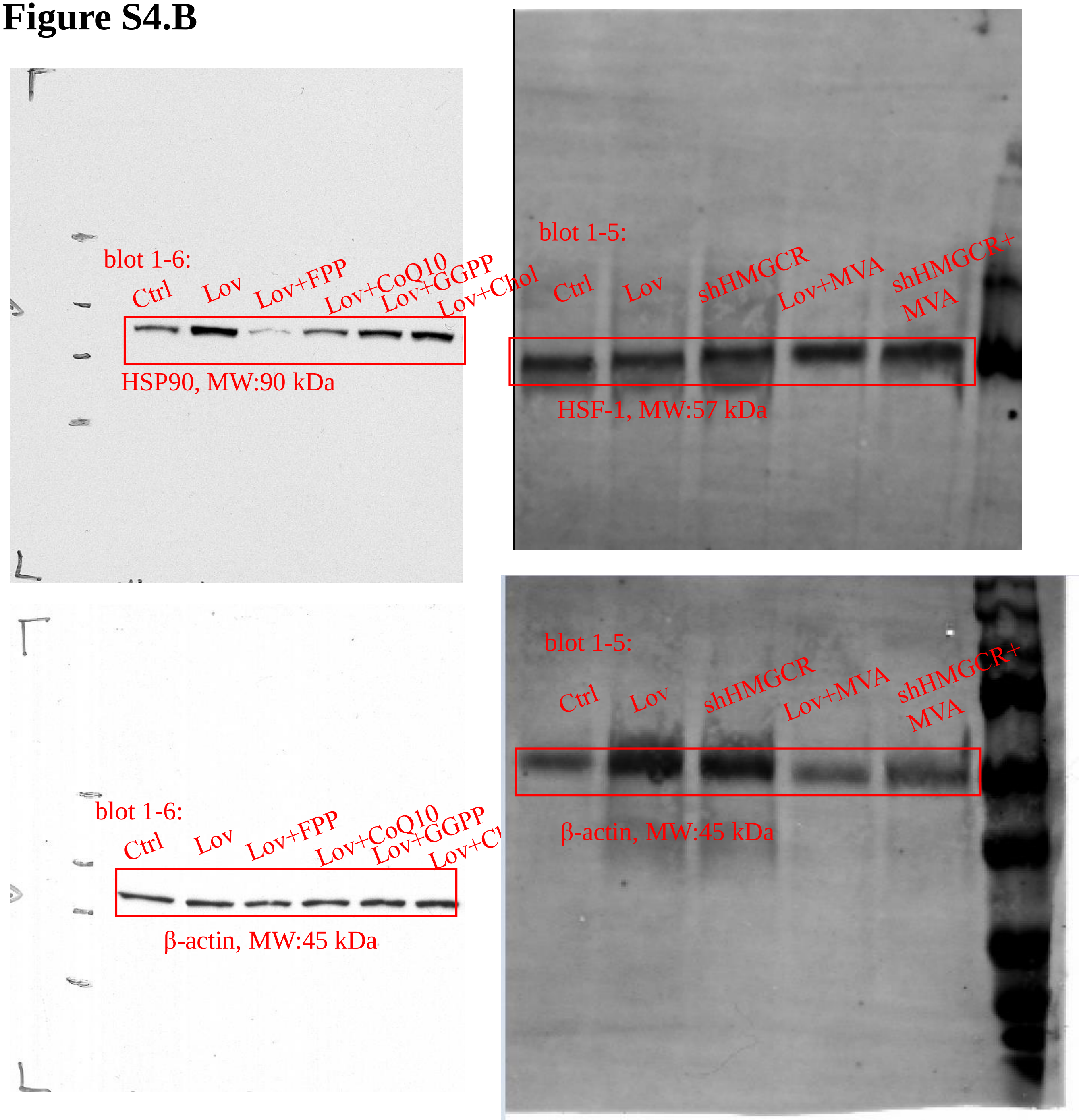

Figure S4.D

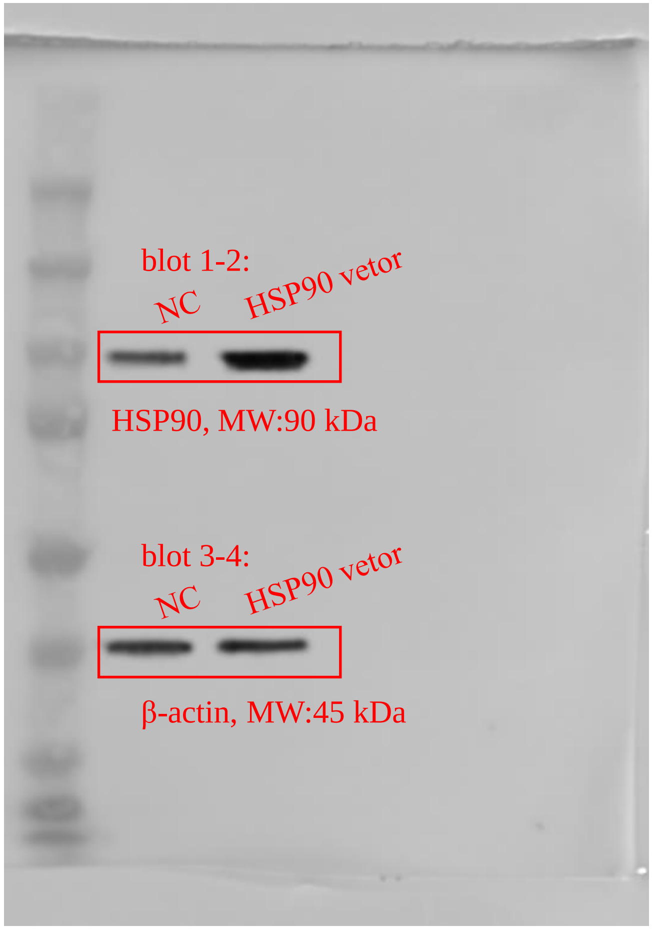

Figure S5.A

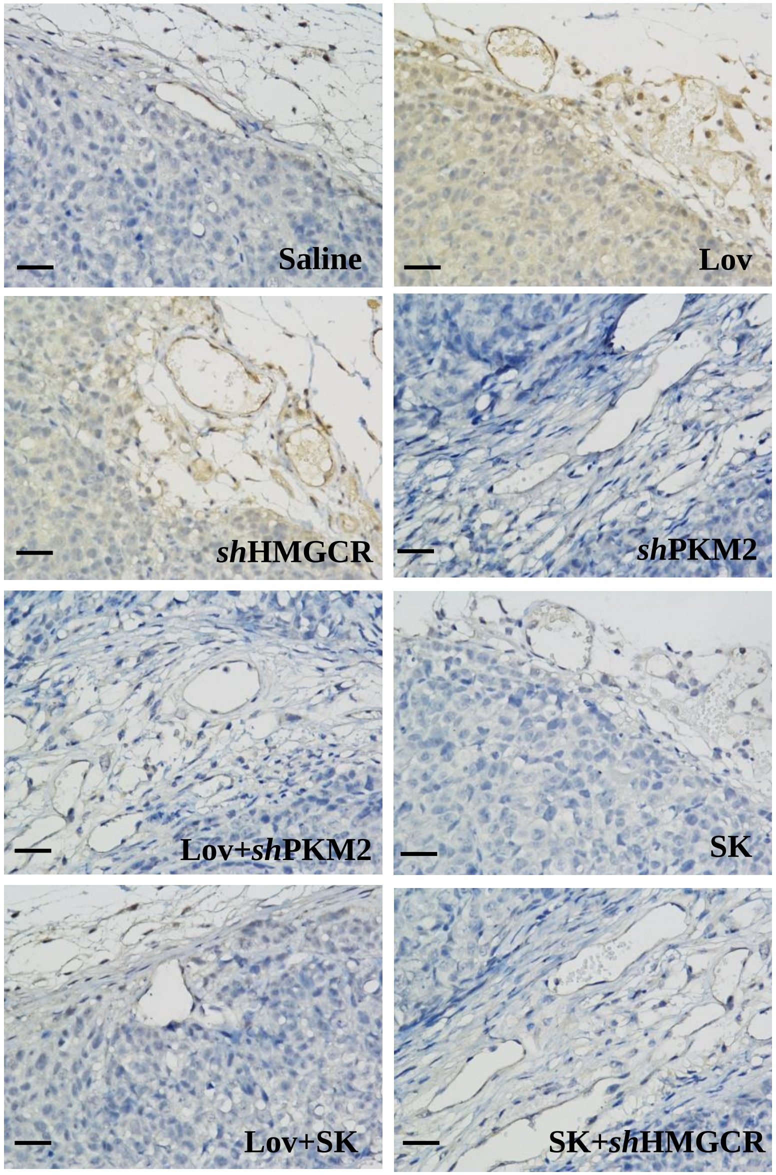

Supplement: S1 Raw Images — (PDF) [file pbio.3001197.s006.pdf]
