# Supplementary material for: HMGCR inhibition stabilizes the glycolytic enzyme PKM2 to support the growth of renal cell carcinoma
Source: PLoS Biol. 2021 Apr 27;19(4):e3001197. doi: 10.1371/journal.pbio.3001197 (PMC8104400; doi:10.1371/journal.pbio.3001197)
Supplement: Attachment — Submitted filename: Response to Reviewer.docx [file pbio.3001197.s007.docx]

**Response to Reviewer #1**

**Overall assessment:**

**This is an interesting study that reports a novel mechanism underlying inhibition of HMCGR accelerating RCC progression via the HSP90/PKM2 axis. I have some comments, questions, and suggestions for the authors to consider which may serve to further strengthen the manuscript:**

**Response:**

We are incredibly grateful to your comments for the manuscript. According with your advice, we amended the relevant part in manuscript. All your questions were answered below.

**Comment 1:**

**“HMGCR inhibition did not directly impact the proliferation of RCC cells *in vitro*, but promotes tumor progression in vivo. Previous studies suggest that cancer cell-produced lactate activates CAFs or TAMs, which in turn promote tumor progression. Please discuss this.”**

**Response:**

Thank you for your suggestion, the accumulation of lactate will indeed regulate CAFs and TAMs. The relevant discussion is as follows, and we have added it to the Discussion section of the manuscript.

“Tumor microenvironment (TME) is the key regulator of carcinogenesis and is composed of many different types of cells, including duo-malignant cancer cells, cancer-associated fibroblasts (CAF), endothelial cells, immune cells, non-cancerous cells, and a variety of peptide factors such as growth factors, cytokines，chemokines and antibodies [1]. The accumulation of lactate in the TME can act as an energy-rich substrate and signal molecule for malignant cancer cells [2]. Acidosis caused by high levels of lactate in the TME can also be beneficial to the metastasis of cancer cells, vascular proliferation, and immunosuppression, thus resulting in a poor clinical prognosis [3]. It has also been shown that high concentrations of lactate found in biopsy tissues from a variety of cancers (kidney, cervical, head and neck, lung, breast, and colorectal cancer) increase the risk of metastasis [4-8]. Moreover, there is a positive correlation between the grade of a cancerous lesion and the concentration of lactate [9]. In addition, as a major component of the TME, cancer-associated fibroblasts (CAFs) can be activated during tumorigenesis and undergo both morphological and functional changes. CAFs produce large amounts of lactate and releases it into the TME. Adjacent cancer cells then absorb this lactate as an alternative energy source to promoting the occurrence, development, and metastasis of tumors [10]. Correspondingly, the lactate produced by tumor cells increases the production of α-ketoglutarate (AKG) in mesenchymal stem cells (MSCs) and promotes the differentiation of MSCs into CAF [11]. Furthermore, tumor-mediated lactate flux is related to the widespread epigenome reprogramming during the formation of CAF [11]. In our study, the concentration of lactate in the tumor cells and tissues of the HMGCR inhibition group were significantly increased; this was positively correlated with the development of transplanted tumors in mice. Furthermore, CD31 immunohistochemistry indicated an increase in abnormal angiogenesis tumors from the HMGCR inhibition group. These data confirm the conclusions of previous studies.”

**Comment 2:**

**“Does HMGCR inhibition affect other glycolysis-related regulators? Such as HK2, GLUT1, and LDHA. Please clarify this.”**

**Response:**

Thanks for your suggestion. We analyzed the others glycolysis-related regulators expression level under HMGCR inhibition, including Hif-1α, akt, c-myc, glut1, HK1/2, enolase1, tigar and LDHA. But the results demonstrated that the expression level of those regulators shown no significant different between with and without HMGCR inhibition. We have added relevant western blot data in Supplementary Figure 2A.

**Comment 3:**

**“Does HMGCR function as a transcription factor? If no, how HMGCR regulate HSP90 transcription in RCC cells? Please explore the potential mechanism.”**

**Response:**

Thanks for your comments. We have not yet obtained direct evidence that HMGCR can act as a transcription factor for HSP90. However, we found that HMGCR inhibition will increase the level of heat shock transcription factor-1 (HSF-1) protein, which may be the reason for the regulation of HSP90. This result has been added to Supplementary Figure 4C. Previous studies have shown that administration of atorvastatin in a human coronary artery endothelial cell model can upregulate HSF-1 protein [12]. Although the underlying mechanisms of HMGCR up-regulates HSF-1 is still unclear, but we will further focus on this in future work.

**Response to Reviewer #2**

**Overall assessment:**

**The manuscript submitted by Huang et al. ("Regulation of 3-Hydroxy-3-methylglutaryl coenzyme A reductase on glycolysis in renal cell carcinoma") describes the role of HMGCR inhibition in promoting tumor growth of renal cell carcinoma. The authors show that loss of HMGCR function by treatment with lovastatin or shRNA upregulated the glycolytic enzyme PKM2, through stabilization by HSP90. Lovastatin treatment increased xenograft tumor growth, but PKM2 inhibition alone or in combination with lovastatin, showed no difference in tumor size. The impact of the mevalonate pathway and statin treatment on cancer development is of great interested, particularly as conflicting reports have demonstrated both tumor promoting and inhibiting roles. The authors use various biochemical assays to convincingly show a relationship between HMGCR inhibition and HSP90-mediated PKM2 regulation in RCC cell lines; however, inclusion of proper controls in many cases makes the current paper unacceptable for publication. It is also very poorly put together and harbors numerous grammatical and typographical errors.**

**Response:**

We thank you for the interests and valuable comments in our study. We have carried out new experiments and added new data in the revised manuscript per your suggestions.

**Comment 1:**

**“The manuscript is poorly written with many grammatical and spelling errors that make the authors' interpretation of the data very difficult to understand. The authors need to extensively revise the writing before the manuscript can be considered for publication anywhere.”**

**Response:**

We polished up the manuscript and corrected many linguistic and incomprehensible places. The expression of the new version manuscript is clearer than before, hoping to make it easier for readers to understand what the author wishes to present.

**Comment 2:**

**“The current title does not accurately describe the work presented in the manuscript, as very few experiments actually explore the regulation of HMGCR and glycolysis (only lactate production and ECAR measurements). A more appropriate title might be something like: "HMGCR inhibition stabilizes glycolytic enzyme PKM2 through HSP90 upregulation to support tumor growth of renal cell carcinoma"”**

**Response:**

Thanks for your suggestions. The manuscript title has been changed to: "HMGCR inhibition stabilizes the glycolytic enzyme PKM2 to support the growth of renal cell carcinoma by upregulating HSP90".

**Comment 3:**

**“Statistical analysis of tumor volume and weight are missing in Figures 1A and 1B.”**

**Response:**

In the revised manuscript, we processed the statistical analysis of tumor volume and weight data.

**Comment 4:**

**“For all usage of shRNAs, please include evidence of knockdown.”**

**Response:**

We have completed all western blot result of *sh*RNA knockdown to the supplementary. And reference of *sh*RNA usage has been cited.

**Comment 5:**

**“In Figure 2D, treatment with lovastatin decreases ECAR while shHMGCR increases ECAR. However, in the results section, this data is described as "ECAR…increased with HMGCR inhibition or knockdown". Can the authors clarify this discrepancy?”**

**Response:**

We are very sorry that we uploaded the unchecked version of Figure and thank you for your reminder. The group label here is incorrect, the blue and brown signs should be interchanged. The actual experimental results are consistent with the description in the manuscript. To confirm this result, we did the experiment again, and the new experiment results are as follows. The data still indicates that the ECAR rate of RCC cells treated with lovastatin increased significantly.

**Reviewer only Figure 1**

**Comment 6:**

**“The authors claim to have "examined the expression levels of glycolysis enzymes and already known upstream regulators". Other than levels of PKM2 in Figure 3, have the expression levels of other glycolytic enzymes upon inhibition of HMGCR been investigated? A previous study by the authors identified HMGCR regulation of lactate in skeletal muscle through enolase modulation (Huang et al. EBioMedicine 2019). Does this hold true in RCC?”**

**Response:**

Thanks for your comments. We also analyzed the others glycolysis-related regulators expression level under HMGCR inhibition, including Hif-1α, akt, c-myc, glut1, HK1/2, enolase1, tigar and LDHA. But the results demonstrated that the expression level of those regulators shown no significant different between with and without HMGCR inhibition. We have added relevant western blot data in Supplementary Figure 2A.

My previous study pointed out that HMGCR inhibition increase the accumulation of lactate in skeletal muscle by regulating ββ-enolase (Enolase 3). However, this regulatory pathway is only applicable to muscle cells and not to RCC cells, because ββ-enolase (Enolase 3) is only specifically expressed in muscle cells. RCC cells express αα-enolase (Enolase 1). The Western blot results of αα-enolase protein showed that treatment of lovastatin or *sh*HMGCR had no effect on it.

**Comment 7:**

**“Figure 3F and Supp Figure 2A are described as "a decrease in the expression of PKM2 protein level resulted in lactate production and glucose consumption decline, and this effect could not be reversed by further application of lovastatin". In order to make this claim, a control of shPKM2 alone must be included in this experiment. Otherwise, the results show that loss of PKM2 completely reverses that induction of lactate production and glucose consumption induced by lovastatin.”**

**Response:**

Corrected accordingly. We have repeated this experiment and added *sh*PKM2 alone experiment groups in the revised manuscript.

**Comment 8:**

**“Related to comment 6- in the writing, the authors mistakenly reference Figure 3E.”**

**Response:**

Corrected accordingly. Figure 3F should be referenced here.

**Comment 9:**

**“Also related to comment 6- what are the comparisons made for the indicated statistics?”**

**Response:**

The Western blot band intensities were quantified using densitometric analysis and normalized to β-actin (Reviewer only Figure 2). The data are expressed as fold changes relative to the DMSO-treated control. Data from the bar chart represent the mean values ± S.D. of three independent experiments. The result shown that there was no significant different with and without HMGCR inhibition for these enzymes.

 **Reviewer only Figure 2**

**Comment 10:**

**“"…exogenous expression of PKM2 significantly increased lactate production.." should reference Figure 3G, not 3H.”**

**Response:**

Corrected accordingly.

**Comment 11:**

**“"consistent with the trend…" describing Figure 3I seems out of place. Please move this description to before the introduction of the CHX experiments.”**

**Response:**

Corrected accordingly.

**Comment 12:**

**“Figure 3K- "… MG132 to rescue the decline of PKM2 induced by mevalonate** **supplementation." In the displayed experiment, the authors have not supplemented with mevalonate. Inclusion of controls including lov alone, shHMGCR alone, and rescues with MVA would greatly strengthen this data.”**

**Response:**

Sorry for the error in the result description. The correct description should be "MG132 rescued the increase in PKM2 caused by the interference of lovastatin and shHMGCR". We have corrected it in the revised manuscript. As you suggested, we added the MVA supplementary experiment as shown below (Reviewer only, Figure 3). The results showed that supplementation of MVA reduced the effect of high concentrations of PKM2 protein induced by HMGCR inhibition. But this effect disappeared under the intervention of MG132. This result still proves that HMGCR inhibition regulates the PKM2 protein concentration by interfering with the protein degradation process.

**Reviewer only Figure 3**

**Comment 13:**

**“"As shown in Figure 5C…" references Figure 4C.”**

**Response:**

Corrected accordingly.

**Comment 14:**

**“Figure 4C: change label to IP: PKM2.”**

**Response:**

Corrected accordingly.

**Comment 15:**

**“Figure 5B: lanes need to be labeled.”**

**Response:**

Corrected accordingly.

**Comment 16:**

**“Figure 5F: Data is not consistent with the rest of Figure 5 or the description of the figure. The +shHSP90 lane has reduced ubiquitination of PKM2. The authors need to include a control blot of HSP90 to confirm loss of HSP90 in this experiment.”**

**Response:**

Thanks for your reminder. Like Comment 5, I apologize again for uploading the unchecked version of Figure. The +/- label of the group here is incorrectly marked. The lane that shHSP90+ group should be the one on the right. The actual experimental results are consistent with the description in the manuscript. To confirm this result, we did the experiment again, and the new experiment results are as follows (Reviewer only Figure 4). The data still indicates that PKM2 protein produced ubiquitination in RCC cells with shHSP90.

**Reviewer only Figure 4**

**Comment 17:**

**“Figure 6: This xenograft experiment should be repeated including untreated tumors (control shRNA, saline only), as well as Lovastatin alone in order to show that any phenotypes are truly "reversed". Additionally, inclusion of shHMGCR tumors with Shikonin treatment would provide additional support to the translational claims made by the authors.”**

**Response:**

Thanks for your suggestions. We already repeated Xenograft experiment including 8 experiment groups (Saline, Lovastatin, *sh*HMGCR, *sh*PKM2, Lov+*sh*PKM2, SK, SK+Lov, SK+*sh*HMGCR) (Figure 6A). Lovastatin and *sh*HMGCR groups will be compared with SHC002 (control shRNA) group to confirm that HMGCR inhibition promotes tumor growth. The remaining groups will be compared with the Lovastatin group and *sh*HMGCR group to confirm the effect of reversed.

**Comment 18:**

**“The authors mention the use of HMGCR as a potential biomarker for treatment with a glycolysis inhibitor. What is the baseline expression of HMGCR, HSP90, and PKM2 in the cell lines used for the experiments? Would inhibition of PKM2, perhaps through** **Shikonin treatment, be more effective in tumors with the lowest levels of HMGCR?”**

**Response:**

Thanks for your comments. The baseline expression of HMGCR, HSP90 and PKM2 in the cell lines used in our study ACHN and 786-O were illustrated in Reviewer only Table1. Several studies have proposed that HMGCR and PKM2 can be used as potential biomarkers to characterize the development of a variety of tumors [13-15]. However, for RCC tumors, the role of HMGCR and PKM2 as biomarkers has not been paid attention. According to the logic of our study, lower HMGCR expression does lead to an increase in PKM2, which makes the treatment of PKM2 inhibitor more effective. In the comparison of others renal cancer cell lines, the expression of HMGCR was the highest in 786-O (79TPM) and the lowest in ACHN (35TPM) (Reviewer only Figure 5). Cell proliferation experiments revealed that ACHN cells with lower HMGCR expression than 786-O cells were indeed inhibited more significantly under the same concentration of Shikonin treatment (Reviewer only Figure 6). However, considering the complexity and variability of the clinical tumor status, this conclusion still needs more clinical data to support.

**Table 1 Baseline expression of HMGCR, HSP90 and PKM2 in ACHN and 786-O**

| **Gene expression (TPM)** | **ACHN** | **786-O** |
| --- | --- | --- |
| **hmgcr** | **35** | **79** |
| **hsp90AA1** | **1858** | **1324** |
| **hsp90AB1** | **3119** | **2776** |
| **pkm2** | **1538** | **1328** |

**Reviewer only Table 1**

**Reviewer only Figure 5**

**Reviewer only Figure 6**

**Comment 19:**

**“What are the levels of HMGCR in primary tumor samples compared to normal kidney tissue, based on TCGA datasets?”**

**Response:**

Thanks for your comments. Available clinical data from the TCGA database indicate that HMGCR levels in primary tumor samples are much lower compared to normal kidney tissue. And we further use the UALCAN website to analyze the HMGCR expression level of RCC patients to perform prognostic analysis [16]. As shown in Reviewer only Figure 7, in kidney renal clear cell carcinoma (KIRC) and kidney renal papillary cell carcinoma (KIRP), the low expression level of HMGCR was significantly associated with the occurrence of primary renal tumors (*p* <0.0001) (Reviewer only Figure 7). This corresponds to the experimental result that HMGCR inhibits the development of RCC. Then, we further analyzed the correlation between HMGCR expression level and patient survival. In KIRC, the most common type of RCC, low expression levels of HMGCR were associated with lower survival rates (*p* = 0.0063) (Reviewer only Figure 8A). But, in KIRP types, which account for approximately 10% to 15% of RCC [17], there is no strong evidence that there is any correlation between HMGCR expression and survival (Reviewer only Figure 8B).

**Reviewer only Figure 7** Expression of HMGCR in primary tumor samples and normal kidney tissue. (A) Kidney renal clear cell carcinoma and (B) kidney renal papillary cell carcinoma.

**Reviewer only Figure 8** Effect of HMGCR expression level on kidney cancer patient survival. (A) Kidney renal clear cell carcinoma (KIRC) and (B) kidney renal papillary cell carcinoma (KIRP).

**Reference**

1.	Lyssiotis CA, Kimmelman AC. Metabolic Interactions in the Tumor Microenvironment. Trends in cell biology. 2017;27(11):863-75. doi: 10.1016/j.tcb.2017.06.003. PubMed PMID: 28734735; PubMed Central PMCID: PMC5814137.

2.	Ippolito L, Morandi A, Giannoni E, Chiarugi P. Lactate: A Metabolic Driver in the Tumour Landscape. Trends Biochem Sci. 2019;44(2):153-66. Epub 2018/11/27. doi: 10.1016/j.tibs.2018.10.011. PubMed PMID: 30473428.

3.	Dhup S, Dadhich RK, Porporato PE, Sonveaux P. Multiple biological activities of lactic acid in cancer: influences on tumor growth, angiogenesis and metastasis. Curr Pharm Des. 2012;18(10):1319-30. Epub 2012/03/01. doi: 10.2174/138161212799504902. PubMed PMID: 22360558.

4.	Girgis H, Masui O, White NM, Scorilas A, Rotondo F, Seivwright A, et al. Lactate dehydrogenase A is a potential prognostic marker in clear cell renal cell carcinoma. Mol Cancer. 2014;13:101. Epub 2014/06/03. doi: 10.1186/1476-4598-13-101. PubMed PMID: 24885701; PubMed Central PMCID: PMCPMC4022787.

5.	Walenta S, Wetterling M, Lehrke M, Schwickert G, Sundfor K, Rofstad EK, et al. High lactate levels predict likelihood of metastases, tumor recurrence, and restricted patient survival in human cervical cancers. Cancer Res. 2000;60(4):916-21. Epub 2000/03/08. PubMed PMID: 10706105.

6.	Brizel DM, Schroeder T, Scher RL, Walenta S, Clough RW, Dewhirst MW, et al. Elevated tumor lactate concentrations predict for an increased risk of metastases in head-and-neck cancer. Int J Radiat Oncol Biol Phys. 2001;51(2):349-53. Epub 2001/09/25. doi: 10.1016/s0360-3016(01)01630-3. PubMed PMID: 11567808.

7.	Vlachostergios PJ, Oikonomou KG, Gibilaro E, Apergis G. Elevated lactic acid is a negative prognostic factor in metastatic lung cancer. Cancer Biomark. 2015;15(6):725-34. Epub 2015/09/26. doi: 10.3233/CBM-150514. PubMed PMID: 26406401.

8.	Walenta S, Schroeder T, Mueller-Klieser W. Lactate in solid malignant tumors: potential basis of a metabolic classification in clinical oncology. Curr Med Chem. 2004;11(16):2195-204. Epub 2004/07/29. doi: 10.2174/0929867043364711. PubMed PMID: 15279558.

9.	Nikoobakht M, Shamshiripour P, Azimi Nekoo Z, Fallah Haghmohammadi S. Elevated Lactate and Total Protein Levels in Stereotactic Brain Biopsy Specimen; Potential Biomarkers of Malignancy and Poor Prognosis. Arch Iran Med. 2019;22(3):125-31. Epub 2019/04/29. PubMed PMID: 31029068.

10.	Whitaker-Menezes D, Martinez-Outschoorn UE, Flomenberg N, Birbe RC, Witkiewicz AK, Howell A, et al. Hyperactivation of oxidative mitochondrial metabolism in epithelial cancer cells in situ: visualizing the therapeutic effects of metformin in tumor tissue. Cell Cycle. 2011;10(23):4047-64. Epub 2011/12/03. doi: 10.4161/cc.10.23.18151. PubMed PMID: 22134189; PubMed Central PMCID: PMCPMC3272287.

11.	Bhagat TD, Von Ahrens D, Dawlaty M, Zou Y, Baddour J, Achreja A, et al. Lactate-mediated epigenetic reprogramming regulates formation of human pancreatic cancer-associated fibroblasts. Elife. 2019;8. Epub 2019/10/31. doi: 10.7554/eLife.50663. PubMed PMID: 31663852; PubMed Central PMCID: PMCPMC6874475.

12.	Li Y, Li J, Cui L, Lai Y, Yao Y, Zhang Y, et al. Inhibitory effect of atorvastatin on AGE-induced HCAEC apoptosis by upregulating HSF-1 protein. Int J Biol Macromol. 2013;57:259-64. Epub 2013/03/21. doi: 10.1016/j.ijbiomac.2013.03.035. PubMed PMID: 23511056.

13.	Bengtsson E, Nerjovaj P, Wangefjord S, Nodin B, Eberhard J, Uhlen M, et al. HMG-CoA reductase expression in primary colorectal cancer correlates with favourable clinicopathological characteristics and an improved clinical outcome. Diagn Pathol. 2014;9:78. Epub 2014/04/09. doi: 10.1186/1746-1596-9-78. PubMed PMID: 24708688; PubMed Central PMCID: PMCPMC4000148.

14.	Sfakianaki M, Papadaki C, Tzardi M, Trypaki M, Manolakou S, Messaritakis I, et al. PKM2 Expression as Biomarker for Resistance to Oxaliplatin-Based Chemotherapy in Colorectal Cancer. Cancers (Basel). 2020;12(8). Epub 2020/07/30. doi: 10.3390/cancers12082058. PubMed PMID: 32722474; PubMed Central PMCID: PMCPMC7465271.

15.	Zhou Z, Li M, Zhang L, Zhao H, Sahin O, Chen J, et al. Oncogenic Kinase-Induced PKM2 Tyrosine 105 Phosphorylation Converts Nononcogenic PKM2 to a Tumor Promoter and Induces Cancer Stem-like Cells. Cancer Res. 2018;78(9):2248-61. Epub 2018/02/15. doi: 10.1158/0008-5472.CAN-17-2726. PubMed PMID: 29440169; PubMed Central PMCID: PMCPMC5932213.

16.	Chandrashekar DS, Bashel B, Balasubramanya SAH, Creighton CJ, Ponce-Rodriguez I, Chakravarthi B, et al. UALCAN: A Portal for Facilitating Tumor Subgroup Gene Expression and Survival Analyses. Neoplasia. 2017;19(8):649-58. Epub 2017/07/22. doi: 10.1016/j.neo.2017.05.002. PubMed PMID: 28732212; PubMed Central PMCID: PMCPMC5516091.

17.	Akhtar M, Al-Bozom IA, Al Hussain T. Papillary Renal Cell Carcinoma (PRCC): An Update. Adv Anat Pathol. 2019;26(2):124-32. Epub 2018/12/07. doi: 10.1097/PAP.0000000000000220. PubMed PMID: 30507616.
