# Supplementary material for: HMGCR inhibition stabilizes the glycolytic enzyme PKM2 to support the growth of renal cell carcinoma
Source: PLoS Biol. 2021 Apr 27;19(4):e3001197. doi: 10.1371/journal.pbio.3001197 (PMC8104400; doi:10.1371/journal.pbio.3001197)
Supplement: Attachment — Submitted filename: Response to Reviewer 2.docx [file pbio.3001197.s008.docx]

**Response to Reviewer #1**

Thank you for your comment.

**Response to Reviewer #2**

**“The authors responded to my concerns well, with one minor exception: the authors claim that they have examined the expression levels of glycolysis enzymes and known upstream regulators (Comment #6) is previous review. They have now assesses a number of upstream regulators (e.g. TIGAR, c-Myc), but still not other enzymes beyond PKM2. The minor edit is that this should be made clear in the text.”**

**Response:**

Thanks for your suggestion. To clarify the point, we changed previous description *“we examined the expression levels of glycolytic enzymes and known upstream regulators (Supplemental Figure 2A) in ACHN cells”* to *“we examined the glycolysis-related regulators expression level under HMGCR inhibition, including Hif-1α, akt, tigar, c-myc, glut1, HK1/2, enolase1, LDHA and PKM2 (Supplemental Figure 2A) in ACHN cells”.*
